# Long-term colorectal cancer incidence after adenoma removal and the effects of surveillance on incidence: a multicentre, retrospective, cohort study

**DOI:** 10.1136/gutjnl-2019-320036

**Published:** 2020-01-17

**Authors:** Amanda J Cross, Emma C Robbins, Kevin Pack, Iain Stenson, Paula L Kirby, Bhavita Patel, Matthew D Rutter, Andrew M Veitch, Brian P Saunders, Stephen W Duffy, Kate Wooldrage

**Affiliations:** 1 Cancer Screening and Prevention Research Group (CSPRG), Department of Surgery and Cancer, Imperial College London, London, UK; 2 Department of Gastroenterology, University Hospital of North Tees, Stockton-on-Tees, UK; 3 Faculty of Medical Sciences, Newcastle University, Newcastle upon Tyne, UK; 4 Department of Gastroenterology, New Cross Hospital, Wolverhampton, UK; 5 Wolfson Unit for Endoscopy, St Mark's Hospital, London, UK; 6 Centre for Cancer Prevention, Wolfson Institute of Preventive Medicine, Queen Mary University of London, London, UK

**Keywords:** adenoma, colonoscopy, colorectal cancer, surveillance

## Abstract

**Objective:**

Postpolypectomy colonoscopy surveillance aims to prevent colorectal cancer (CRC). The 2002 UK surveillance guidelines define low-risk, intermediate-risk and high-risk groups, recommending different strategies for each. Evidence supporting the guidelines is limited. We examined CRC incidence and effects of surveillance on incidence among each risk group.

**Design:**

Retrospective study of 33 011 patients who underwent colonoscopy with adenoma removal at 17 UK hospitals, mostly (87%) from 2000 to 2010. Patients were followed up through 2016. Cox regression with time-varying covariates was used to estimate effects of surveillance on CRC incidence adjusted for patient, procedural and polyp characteristics. Standardised incidence ratios (SIRs) compared incidence with that in the general population.

**Results:**

After exclusions, 28 972 patients were available for analysis; 14 401 (50%) were classed as low-risk, 11 852 (41%) as intermediate-risk and 2719 (9%) as high-risk. Median follow-up was 9.3 years. In the low-risk, intermediate-risk and high-risk groups, CRC incidence per 100 000 person-years was 140 (95% CI 122 to 162), 221 (195 to 251) and 366 (295 to 453), respectively. CRC incidence was 40%–50% lower with a single surveillance visit than with none: hazard ratios (HRs) were 0.56 (95% CI 0.39 to 0.80), 0.59 (0.43 to 0.81) and 0.49 (0.29 to 0.82) in the low-risk, intermediate-risk and high-risk groups, respectively. Compared with the general population, CRC incidence without surveillance was similar among low-risk (SIR 0.86, 95% CI 0.73 to 1.02) and intermediate-risk (1.16, 0.97 to 1.37) patients, but higher among high-risk patients (1.91, 1.39 to 2.56).

**Conclusion:**

Postpolypectomy surveillance reduces CRC risk. However, even without surveillance, CRC risk in some low-risk and intermediate-risk patients is no higher than in the general population. These patients could be managed by screening rather than surveillance.

**Significance of this study**

**What is already known on this subject?**

- Patients thought to be at increased risk of colorectal cancer (CRC) after adenoma removal are recommended surveillance by colonoscopy.
- The 2002 UK surveillance guidelines stratify patients with adenomas into low-risk, intermediate-risk and high-risk groups according to baseline adenoma characteristics, and recommend different surveillance strategies for each risk group.
- The evidence supporting the guidelines is limited. Most studies on adenoma surveillance predate improvements in colonoscopy quality and examine detection rates of advanced adenomas at follow-up colonoscopy rather than long-term risk of CRC.
- Adenoma surveillance currently accounts for 20% of colonoscopies performed in the UK, placing enormous pressure on endoscopy services.
- Some patients may require less surveillance than currently recommended; however, this is uncertain due to the lack of high-quality data with CRC as the outcome.

**Significance of this study**

**What are the new findings?**

- Among ~30 000 patients with adenomas, CRC risk remained elevated in only a third after baseline colonoscopy and polypectomy.
- Patients remaining at increased CRC risk included the whole high-risk group, in addition to intermediate-risk patients with an incomplete colonoscopy, adenoma with high-grade dysplasia or proximal polyps at baseline. Colonoscopy surveillance significantly reduced CRC risk in these patients.
- Colonoscopy surveillance also reduced CRC risk among the remaining two-thirds of patients, including the whole low-risk group and intermediate-risk patients with a complete baseline colonoscopy and no high-grade dysplasia or proximal polyps. However, even without surveillance, these patients had a CRC risk no higher than the general population.

**How might it impact on clinical practice in the foreseeable future?**

- Following a complete baseline colonoscopy and polypectomy, low-risk patients and intermediate-risk patients with no high-grade dysplasia or proximal polyps could be managed by routine screening rather than by surveillance.
- In England, if these patients forewent surveillance and instead returned to the National Health Service Bowel Cancer Screening Programme, numbers of surveillance colonoscopies could be reduced by a third.
- Patients returning to screening should be reminded to see their general practitioner if they experience any lower gastrointestinal symptoms.
- Additional long-term studies and economic evaluations are needed to define optimal surveillance strategies for those remaining at increased CRC risk following adenoma removal.

## Introduction

Colorectal cancer (CRC) causes considerable morbidity and mortality.^1^ It can be prevented by removing adenomas, known precursors.^2^ Patients at increased risk of CRC following adenoma removal are recommended surveillance colonoscopy. The 2002 UK surveillance guidelines stratify patients with adenomas into three risk groups,^3^ as do the European Union (EU) and US guidelines.^4 5^ Low-risk patients (with 1–2 adenomas <10 mm) are recommended no surveillance or surveillance at 5–10 years; while intermediate-risk/higher-risk patients (with 3–4 adenomas <10 mm or 1–2 adenomas with at least 1≥10 mm (UK/EU), or 3–10 adenomas or at least 1≥10 mm, with villous histology, or high-grade dysplasia (US)) are recommended 3-yearly surveillance. High-risk patients (with 5 or more adenomas <10 mm, or 3 or more adenomas with at least 1≥10 mm (UK), or more than 10 adenomas (US)) are recommended colonoscopy at 1 year or within 3 years before 3-yearly surveillance.

The 2002 UK guidelines were largely based on studies using detection rates of advanced adenomas (AAs) at follow-up as a proxy for CRC,^3 6–9^ which overestimates risk due to higher rates of AAs than CRC.^9 10^ Moreover, as the guidelines were developed before substantial improvements in colonoscopy quality,^11^ such intensive surveillance may no longer be necessary.

In 2004, there was a call for proposals to reassess surveillance requirements among intermediate-risk patients, who account for most surveillance colonoscopies.^12^ There was concern that the introduction of the Bowel Cancer Screening Programme (BCSP) in 2006 would increase demand for surveillance and overwhelm endoscopy services. We developed a study that examined CRC incidence among intermediate-risk patients over a median of 7.9 years, identifying a higher-risk subgroup who benefited from surveillance and a lower-risk subgroup who could potentially forego surveillance.^13^

These findings were timely as adenoma surveillance accounts for 20% of colonoscopies performed in the UK and USA, placing great pressure on endoscopy services.^14 15^ Revision of the guidelines is required to minimise unnecessary colonoscopies while ensuring that patients at increased CRC risk receive surveillance. The present study examined CRC incidence among all three risk groups over a median of 9.3 years and assessed effects of surveillance on CRC incidence. We aimed to identify patient subgroups who could safely forego surveillance or receive less than currently recommended.

## Methods

### Study design and participants

We conducted a retrospective study using data from 17 UK hospitals on patients who had adenomas removed at baseline colonoscopy from 1984 to 2010 (mostly (87%) from 2000 to 2010). We used this cohort for our previous study of intermediate-risk patients.^13 16^ For the present study, we obtained updated information on the cohort (eg, on surveillance examinations, cancers and deaths). This provided longer-term follow-up data for the intermediate-risk group. We additionally examined the low-risk and high-risk groups not previously analysed.

Participating hospitals were required to have lower gastrointestinal endoscopy and pathology reports recorded electronically for at least 6 years prior to study start (2006). We searched endoscopy databases for patients who had undergone colonic examination before 31 December 2010, and searched pathology databases for reports of colorectal lesions. Endoscopy and pathology reports were pseudonymised and entered into a database (Oracle Corporation, Redwood City, California, USA). Summary values for size, histology and location were assigned to lesions seen at multiple examinations.^16^

After identifying patients with colonic examinations before 31 December 2010, we looked back in these patients’ records to identify the first occurrence of an adenoma, defining this as baseline. Multiple examinations were sometimes required at baseline to fully examine the colon and remove detected lesions, which we grouped and defined as the baseline visit. Baseline visits sometimes spanned days or months. Subsequent colonic examinations were grouped into surveillance visits, using rules described elsewhere.^16^

We excluded patients without a colonoscopy or adenoma at baseline. We also excluded patients with CRC; a prior bowel resection; inflammatory bowel disease; polyposis, juvenile polyps, or hamartomatous polyps; Lynch syndrome or family history of familial adenomatous polyposis; colorectal carcinoma in situ reported more than 3 years before baseline; missing examination dates; or missing information needed for risk categorisation.

Following the 2002 UK guidelines,^3^ we classed patients into low-risk (1-2 adenomas <10 mm); intermediate-risk (3-4 adenomas <10 mm, or 1-2 adenomas with at least 1 ≥10 mm); and high-risk groups (5 or more adenomas <10 mm, or 3 or more adenomas with at least 1 ≥10 mm).

We obtained data on cancers and deaths from National Health Service (NHS) Digital, NHS Central Register, and National Services Scotland through 2016 and entered these into the study database. We compared the cancer data with the hospital data and resolved data duplication and inconsistency issues.

The primary outcome was incident adenocarcinoma of the colorectum, including cancers with unspecified morphology but assumed to be adenocarcinomas (those located between the rectum and caecum). In situ cancers and cancers with unspecified morphology but assumed to be squamous cell carcinomas (those located around the anus) were not included as CRCs.

In line with previous methodology,^13 16^ we excluded CRCs that we assumed had arisen from incompletely resected baseline lesions because we thought their inclusion could lead to biased estimates of risk and inappropriate surveillance recommendations. Namely, we excluded CRCs found in the same/adjacent colonic segment to a baseline adenoma ≥15 mm that was seen at least twice within 5 years preceding cancer diagnosis. In sensitivity analyses, we additionally excluded CRCs that satisfied only some of these criteria, but that we deemed likely to have arisen from incompletely resected lesions.

### Statistical analysis

Sample size calculations were based on obtaining estimates of CRC incidence with a coefficient of variation of ~30%. Assuming an incidence rate of two CRCs per 1000 person-years,^17–19^ nine CRCs and 4500 person-years in any risk subgroup would give a coefficient of variation of 33%. Thus, assuming the smallest subgroup would be 15% the size of the whole risk group, 60 CRCs were required in each risk group.

We compared baseline characteristics among patients with and without surveillance using χ² tests, including sex, age, adenoma number, size, histology, and dysplasia, presence of proximal polyps, colonoscopy completeness, bowel preparation quality, year of baseline visit, length of baseline visit (in days or months), family history of cancer/CRC, number of hyperplastic polyps and presence of hyperplastic polyps ≥10 mm. Colonoscopy completeness and bowel preparation quality were defined by the most complete colonoscopy and best preparation during baseline.

We estimated CRC incidence after baseline in each risk group. Time-at-risk started from the last examination at baseline. Time-to-event data were censored at first CRC diagnosis, death, emigration or date of complete ascertainment of cases in cancer registries.

We examined effects of surveillance and baseline characteristics on CRC incidence. Exposure to successive surveillance visits started at the last examination in each visit. When CRC was diagnosed at a surveillance visit, we did not include the visit as surveillance as it offered no protection against CRC. We used univariable Cox proportional-hazards models to calculate hazard ratios (HRs) and 95% confidence intervals (CIs). Multivariable Cox regression was used to identify independent CRC risk factors, using backward stepwise selection based on likelihood ratio tests to retain variables with p values <0.05. Number of surveillance visits was included as a time-varying covariate. Interactions between number of surveillance visits and age or sex were assessed by including interaction parameters.

We performed Kaplan-Meier analyses to show time to cancer diagnosis and estimate cumulative CRC incidence with 95% CIs at 3 years, 5 years and 10 years. Cumulative incidence curves were compared using the log-rank test. We calculated standardised incidence ratios (SIRs) as the ratio of observed to expected CRC cases, with exact Poisson 95% CIs. Expected cases were calculated by multiplying sex-specific and 5-year age-group-specific person-years by the corresponding incidence in the general population of England in 2007.^20^ We divided each patient’s follow-up time into distinct periods; in the absence of surveillance, censoring at first surveillance; after first surveillance, censoring at second surveillance; and after second surveillance to final censoring.

Using baseline risk factors, we stratified each risk group into lower-risk and higher-risk subgroups. Age was not included in the stratification criteria because older age is associated with worse colonoscopy quality and higher risks of complications;^21^ nor was year or length of baseline visit which do not help define clinically relevant subgroups.

In our previous study of intermediate-risk patients, incomplete colonoscopies, colonoscopies of unknown completeness, poor bowel preparation, adenomas ≥20 mm, adenomas with high-grade dysplasia and proximal polyps were CRC risk factors.^13 16^ In the present study, we used these factors to define higher-risk in a sensitivity analysis of the risk stratification criteria for intermediate-risk patients. Further sensitivity analyses excluded patients without a complete baseline colonoscopy.

Analyses were conducted in Stata/IC V.13.1 (StataCorp LP, 2013; Stata Statistical Software: Release 13; College Station, Texas, USA). The study is registered (ISRCTN15213649). The protocol is available online.^22^

### Patient and public involvement

Our patient and public representatives reviewed the study proposal and results and have helped to develop plans for wider dissemination of the results.

## Results

**Figure 1:**
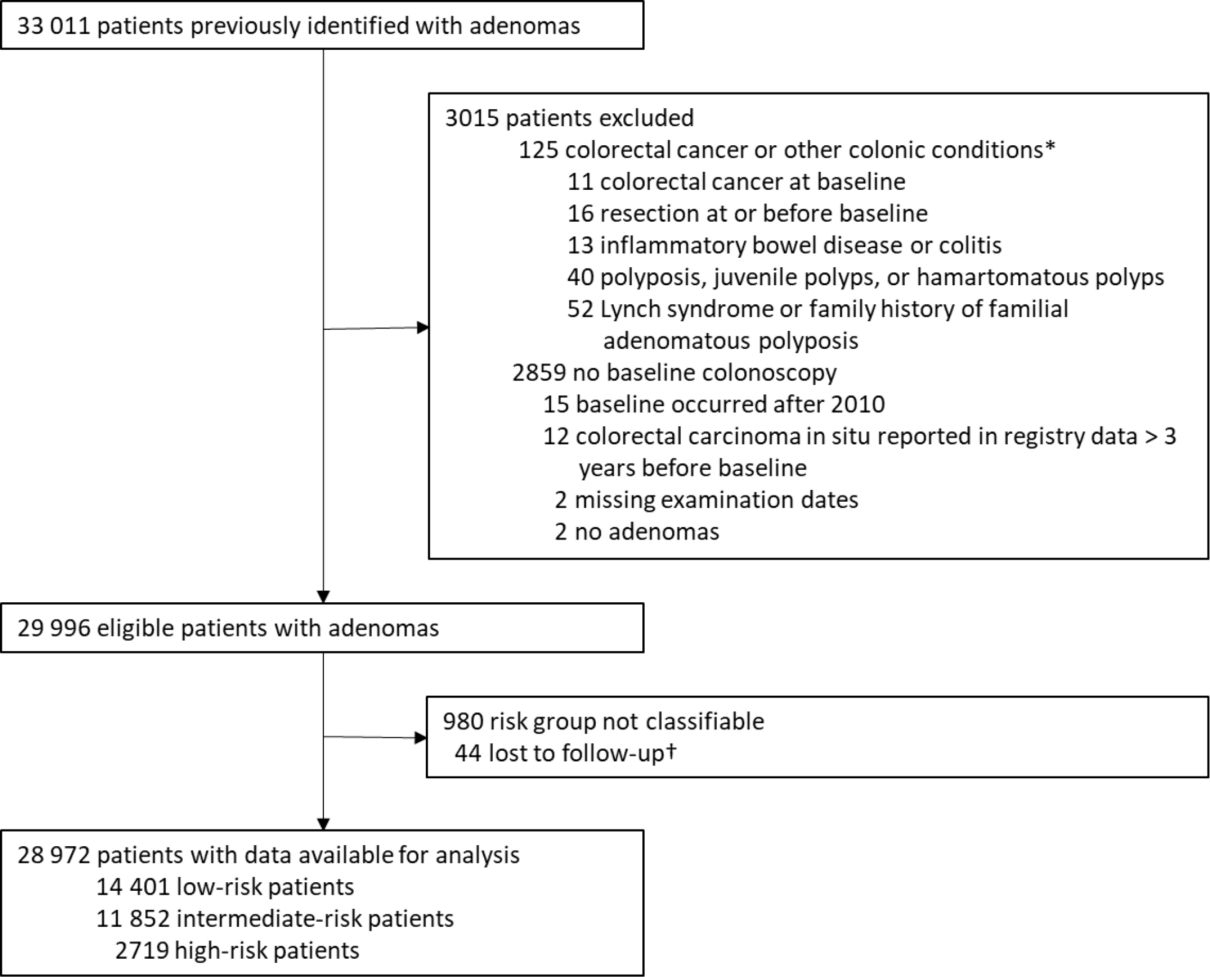
Study profile.*Not mutually exclusive. †Reasons for lost to follow-up: 19 patients had all examinations after emigrating; 22 patients were untraceable through national data sources and had no surveillance; and 3 patients had an unknown date of birth.

There were 33 011 eligible patients in the updated cohort. Of these, we excluded 2859 with no baseline colonoscopy; 125 with CRC at baseline or a condition associated with increased CRC risk; 15 whose baseline occurred after 2010; 12 with colorectal carcinoma in situ more than 3 years before baseline; 2 with missing examination dates; 2 without adenomas; 980 whose risk could not be classified; and 44 who were lost to follow-up. Of the remaining 28 972, 14 401 (50%) were classed as low-risk, 11 852 (41%) as intermediate-risk and 2719 (9%) as high-risk (figure 1).

Patients attending surveillance were younger than non-attenders and generally more likely to have had more adenomas, an adenoma with tubulovillous histology or high-grade dysplasia, hyperplastic polyps or missing data at baseline. A greater proportion of attenders than non-attenders had a baseline visit before 2005, a baseline visit spanning more than 1 day and a family history of cancer/CRC. Non-attenders were more likely to have had an incomplete colonoscopy or poor bowel preparation. Among intermediate-risk patients, attenders were more likely to be male and have had an adenoma ≥20 mm or hyperplastic polyp ≥10 mm (online supplementary table 1).

10.1136/gutjnl-2019-320036.supp1 Supplementary data

The median age of low-risk patients was 64 years (IQR 55 to 72), 44% were women and 50% attended surveillance (table 1). The median time to first surveillance was 3.2 years (IQR 2.2 to 5.0). During a median follow-up of 9.6 years (IQR 7.2 to 12.4), 195 CRCs were diagnosed, giving an incidence rate of 140 per 100 000 person-years (table 1). Number of surveillance visits, age, adenoma histology, proximal polyps and colonoscopy completeness were independently associated with CRC incidence. Adjusting for these factors, a single surveillance visit was associated with a 44% reduction in CRC incidence compared with no surveillance. Incidence was even lower with two surveillance visits (table 1).

**Table 1:** Long-term colorectal cancer (CRC) incidence in the low-risk group by baseline characteristics and number of surveillance visits

	N (%)	Person-years	CRC cases	Incidence per 100 000 person-years (95% CI)	Univariable HR (95% CI)	P value	Multivariable HR (95% CI)	P value
Total	14 401 (100)	138 903	195	140 (122 to 162)	–	–	–	–
Number of surveillance visits*	–	–	–	–	–	<0.0001	–	<0.0001
0	7207 (50.1)	84 591	143	169 (143 to 199)	1	–	1	–
1	3959 (27.5)	34 507	41	119 (87 to 161)	0.55 (0.39 to 0.79)	–	0.56 (0.39 to 0.80)	–
2	1943 (13.5)	12 986	8	62 (31 to 123)	0.26 (0.12 to 0.54)	–	0.27 (0.13 to 0.56)	–
≥3	1292 (9.0)	6818	3	44 (14 to 136)	0.17 (0.05 to 0.57)	–	0.18 (0.05 to 0.58)	–
Sex	–	–	–	–	–	0.67	–	0.99
Women	6382 (44.3)	63 337	92	145 (118 to 178)	1	–	1	–
Men	8019 (55.7)	75 567	103	136 (112 to 165)	0.94 (0.71 to 1.25)	–	1.00 (0.75 to 1.32)	–
Age, years	–	–	–	–	–	<0.0001	–	<0.0001
<55	3569 (24.8)	40 422	21	52 (34 to 80)	1	–	1	–
55–64	3991 (27.7)	42 121	46	109 (82 to 146)	2.12 (1.26 to 3.55)	–	2.05 (1.22 to 3.44)	–
65–74	4258 (29.6)	38 799	76	196 (156 to 245)	3.87 (2.38 to 6.28)	–	3.52 (2.17 to 5.73)	–
≥75	2583 (17.9)	17 561	52	296 (226 to 389)	6.12 (3.67 to 10.20)	–	5.02 (3.00 to 8.39)	–
Number of adenomas	–	–	–	–	–	0.30	–	0.69
1	11 762 (81.7)	113 729	154	135 (116 to 159)	1	–	1	–
2	2639 (18.3)	25 175	41	163 (120 to 221)	1.20 (0.85 to 1.70)	–	1.07 (0.76 to 1.53)	–
Adenoma histology†	–	–	–	–	–	0.0093	–	0.0067
Tubular	11 138 (77.3)	107 018	132	123 (104 to 146)	1	–	1	–
Tubulovillous	2113 (14.7)	20 130	44	219 (163 to 294)	1.69 (1.21 to 2.37)	–	1.71 (1.21 to 2.40)	–
Villous	190 (1.3)	1906	2	105 (26 to 420)		–		–
Unknown	960 (6.7)	9849	17	173 (107 to 278)	1.39 (0.84 to 2.30)	–	1.52 (0.92 to 2.52)	–
Adenoma dysplasia†	–	–	–	–	–	0.0543	–	0.0775
Low-grade	13 242 (92.0)	125 812	171	136 (117 to 158)	1	–	1	–
High-grade	357 (2.5)	3469	11	317 (176 to 573)	2.32 (1.26 to 4.28)	–	2.20 (1.18 to 4.10)	–
Unknown	802 (5.6)	9623	13	135 (78 to 233)	0.99 (0.56 to 1.74)	–	0.93 (0.52 to 1.66)	–
Proximal polyps	–	–	–	–	–	0.0046	–	0.002
No	8133 (56.5)	80 118	93	116 (95 to 142)	1	–	1	–
Yes	6268 (43.5)	58 785	102	174 (143 to 211)	1.50 (1.13 to 1.99)	–	1.57 (1.18 to 2.10)	–
Completeness of colonoscopy	–	–	–	–	–	0.17	–	0.0274
Complete	11 719 (81.4)	108 319	144	133 (113 to 157)	1	–	1	–
Incomplete	1140 (7.9)	10 674	26	244 (166 to 358)	1.26 (0.91 to 1.74)	–	1.47 (1.05 to 2.04)	–
Unknown	1542 (10.7)	19 910	25	126 (85 to 186)		–		–
Bowel preparation quality	–	–	–	–	–	0.15	–	0.32
Excellent or good	5145 (35.7)	52 129	84	161 (130 to 200)	1	–	1	–
Satisfactory	2540 (17.6)	22 051	30	136 (95 to 195)	0.85 (0.56 to 1.29)	–	0.81 (0.53 to 1.23)	–
Poor	968 (6.7)	7970	15	188 (113 to 312)	1.18 (0.68 to 2.04)	–	1.09 (0.63 to 1.88)	–
Unknown	5748 (39.9)	56 754	66	116 (91 to 148)	0.72 (0.52 to 1.00)	–	0.76 (0.55 to 1.05)	–
Year of baseline visit	–	–	–	–	–	0.71	–	0.45
1984–1994	1640 (11.4)	23 185	32	138 (98 to 195)	1	–	1	–
2000–2004	5168 (35.9)	56 134	86	153 (124 to 189)	1.06 (0.69 to 1.62)	–	0.93 (0.60 to 1.43)	–
2005–2010	7593 (52.7)	59 585	77	129 (103 to 162)	0.92 (0.59 to 1.44)	–	0.77 (0.48 to 1.23)	–
Length of baseline visit	–	–	–	–	–	0.74	–	0.64
1 day	11 354 (78.8)	110 143	152	138 (118 to 162)	1	–	1	–
2 days–3 months	1373 (9.5)	12 314	19	154 (98 to 242)	1.12 (0.70 to 1.81)	–	1.13 (0.70 to 1.84)	–
3–6 months	950 (6.6)	9309	16	172 (105 to 281)	1.24 (0.74 to 2.07)	–	1.39 (0.82 to 2.33)	–
≥6 months	724 (5.0)	7137	8	112 (56 to 224)	0.81 (0.40 to 1.65)	–	0.91 (0.45 to 1.87)	–

The final multivariable model contained number of surveillance visits, age, adenoma histology, proximal polyps and completeness of colonoscopy. For these variables, the multivariable HRs were from the final multivariable model and the p values were for inclusion of the variable in the model, calculated with the likelihood ratio test. For the remaining variables, the multivariable HRs were for if the variable was added as an additional variable to the final multivariable model.

*The number of surveillance visits was included as a time-varying covariate; patients who attended any surveillance contributed person-years to more than one category of number of surveillance visits.

†Adenoma histology and dysplasia were defined according to the worst histology (greatest degree of villousness) and worst dysplasia (highest grade) seen at baseline, respectively.

The median age of intermediate-risk patients was 66 years (IQR 58 to 74), 44% were women and 60% attended surveillance (table 2). The median time to first surveillance was 3.0 years (IQR 1.4 to 3.5). During a median follow-up of 9.1 years (IQR 6.6 to 12.4), 246 CRCs were diagnosed, giving an incidence rate of 221 per 100 000 person-years (table 2). Number of surveillance visits, age, adenoma dysplasia, proximal polyps, colonoscopy completeness, and year and length of baseline visit were independently associated with CRC incidence. Adenoma histology was not included in the final multivariable model because it was only associated with incidence when the unknown category was included. Adjusting for the other factors, a single surveillance visit was associated with a 41% reduction in CRC incidence compared with no surveillance. A similar reduction in incidence was seen with two surveillance visits (table 2).

**Table 2:** Long-term colorectal cancer (CRC) incidence in the intermediate-risk group by baseline characteristics and number of surveillance visits

	N (%)	Person-years	CRC cases	Incidence per 100 000 person-years (95% CI)	Univariable HR (95% CI)	P value	Multivariable HR (95% CI)	P value
Total	11 852 (100)	111 270	246	221 (195 to 251)	–	–	–	–
Number of surveillance visits*	–	–	–	–	–	0.0004	–	0.0009
0	4683 (39.5)	53 927	135	250 (211 to 296)	1	–	1	–
1	3343 (28.2)	33 284	62	186 (145 to 239)	0.58 (0.42 to 0.79)	–	0.59 (0.43 to 0.81)	–
2	2279 (19.2)	15 477	31	200 (141 to 285)	0.53 (0.35 to 0.81)	–	0.56 (0.36 to 0.85)	–
≥3	1547 (13.1)	8582	18	210 (132 to 333)	0.44 (0.26 to 0.77)	–	0.45 (0.26 to 0.77)	–
Sex	–	–	–	–	–	0.28	–	0.0549
Women	5271 (44.5)	51 049	105	206 (170 to 249)	1	–	1	–
Men	6581 (55.5)	60 221	141	234 (199 to 276)	1.15 (0.89 to 1.48)	–	1.28 (0.99 to 1.66)	–
Age, years	–	–	–	–	–	<0.0001	–	<0.0001
<55	2097 (17.7)	24 995	28	112 (77 to 162)	1	–	1	–
55–64	3158 (26.7)	33 530	52	155 (118 to 204)	1.44 (0.91 to 2.28)	–	1.41 (0.89 to 2.24)	–
65–74	3915 (33.0)	35 391	98	277 (227 to 338)	2.74 (1.80 to 4.19)	–	2.66 (1.74 to 4.06)	–
≥75	2682 (22.6)	17 354	68	392 (309 to 497)	4.25 (2.71 to 6.65)	–	3.64 (2.31 to 5.74)	–
Number of adenomas	–	–	–	–	–	0.37	–	0.20
1	7793 (65.8)	74 791	168	225 (193 to 261)	1	–	1	–
2	3053 (25.8)	27 502	64	233 (182 to 297)	1.06 (0.79 to 1.41)	–	0.92 (0.68 to 1.25)	–
3 or 4	1006 (8.5)	8977	14	156 (92 to 263)	0.71 (0.41 to 1.23)	–	0.61 (0.34 to 1.08)	–
Adenoma size, mm†	–	–	–	–	–	0.0866	–	0.18
<10	1006 (8.5)	8977	14	156 (92 to 263)	1	–	1	–
10–19	6802 (57.4)	64 716	134	207 (175 to 245)	1.30 (0.75 to 2.26)	–	1.53 (0.87 to 2.70)	–
≥20	4044 (34.1)	37 577	98	261 (214 to 318)	1.64 (0.94 to 2.88)	–	1.69 (0.94 to 3.04)	–
Adenoma histology‡	–	–	–	–	–	<0.0001	–	0.0025
Tubular	4694 (39.6)	44 369	71	160 (127 to 202)	1	–	1	–
Tubulovillous	5537 (46.7)	51 211	114	223 (185 to 267)	1.40 (1.04 to 1.88)	–	1.29 (0.95 to 1.75)	–
Villous	1134 (9.6)	10 108	31	307 (216 to 436)	1.93 (1.27 to 2.95)	–	1.44 (0.93 to 2.24)	–
Unknown	487 (4.1)	5581	30	538 (376 to 769)	3.23 (2.10 to 4.98)	–	2.76 (1.64 to 4.64)	–
Adenoma dysplasia‡	–	–	–	–	–	0.0002	–	0.0038
Low-grade	9399 (79.3)	87 581	166	190 (163 to 221)	1	–	1	–
High-grade	1979 (16.7)	17 402	53	305 (233 to 399)	1.62 (1.19 to 2.21)	–	1.47 (1.07 to 2.02)	–
Unknown	474 (4.0)	6287	27	429 (295 to 626)	2.11 (1.39 to 3.20)	–	1.86 (1.21 to 2.86)	–
Proximal polyps	–	–	–	–	–	0.0284	–	0.0025
No	8254 (69.6)	79 798	162	203 (174 to 237)	1	–	1	–
Yes	3598 (30.4)	31 471	84	267 (216 to 331)	1.35 (1.04 to 1.76)	–	1.54 (1.17 to 2.02)	–
Completeness of colonoscopy	–	–	–	–	–	0.0007	–	0.0022
Complete	8967 (75.7)	80 572	150	186 (159 to 218)	1		1	–
Incomplete	1321 (11.2)	11 545	49	424 (321 to 562)	1.58 (1.22 to 2.06)	–	1.55 (1.18 to 2.06)	–
Unknown	1564 (13.2)	19 152	47	245 (184 to 327)		–		–
Bowel preparation quality	–	–	–	–	–	0.13	–	0.14
Excellent or good	3974 (33.5)	37 493	71	189 (150 to 239)	1	–	1	–
Satisfactory	1903 (16.1)	15 451	36	233 (168 to 323)	1.28 (0.86 to 1.92)	–	1.47 (0.98 to 2.22)	–
Poor	660 (5.6)	4840	17	351 (218 to 565)	1.92 (1.13 to 3.25)	–	1.67 (0.98 to 2.85)	–
Unknown	5315 (44.8)	53 485	122	228 (191 to 272)	1.17 (0.88 to 1.57)	–	1.13 (0.84 to 1.53)	–
Year of baseline visit	–	–	–	–	–	0.0044	–	0.0078
1984–1999	1870 (15.8)	25 329	83	328 (264 to 406)	1	–	1	–
2000–2004	4222 (35.6)	42 957	92	214 (175 to 263)	0.66 (0.48 to 0.90)	–	0.63 (0.46 to 0.87)	–
2005–2010	5760 (48.6)	42 983	71	165 (131 to 208)	0.57 (0.40 to 0.80)	–	0.59 (0.40 to 0.85)	–
Length of baseline visit	–	–	–	–	–	0.0181	–	0.0082
1 day	6697 (56.5)	63 453	117	184 (154 to 221)	1	–	1	–
2 days–3 months	2343 (19.8)	21 669	60	277 (215 to 357)	1.53 (1.12 to 2.08)	–	1.65 (1.20 to 2.26)	–
3–6 months	1403 (11.8)	13 277	32	241 (170 to 341)	1.32 (0.89 to 1.95)	–	1.34 (0.90 to 1.99)	–
≥6 months	1409 (11.9)	12 871	37	287 (208 to 397)	1.57 (1.09 to 2.27)	–	1.58 (1.08 to 2.30)	–

The final multivariable model contained number of surveillance visits, age, adenoma dysplasia, proximal polyps, completeness of colonoscopy, year of baseline visit and length of baseline visit. For these variables, the multivariable HRs were from the final multivariable model and the p values were for inclusion of the variable in the model, calculated with the likelihood ratio test. For the remaining variables, the multivariable HRs were for if the variable was added as an additional variable to the final multivariable model.

*The number of surveillance visits was included as a time-varying covariate; patients who attended any surveillance contributed person-years to more than one category of number of surveillance visits.

†Adenoma size was defined according to the largest adenoma seen at baseline.

‡Adenoma histology and dysplasia were defined according to the worst histology (greatest degree of villousness) and worst dysplasia (highest grade) seen at baseline, respectively.

The median age of high-risk patients was 67 years (IQR 61 to 74), 29% were women and 66% attended surveillance (table 3). The median time to first surveillance was 1.5 years (IQR 1.0 to 3.0). During a median follow-up of 8.4 years (IQR 5.7 to 11.2), 84 CRCs were diagnosed, giving an incidence rate of 366 per 100 000 person-years (table 3). Number of surveillance visits, adenoma dysplasia and colonoscopy completeness were independently associated with CRC incidence. Adjusting for these factors, a single surveillance visit was associated with a halving of CRC incidence compared with no surveillance. Attendance at subsequent visits was associated with further incidence reductions (table 3).

**Table 3:** Long-term colorectal cancer (CRC) incidence in the high-risk group by baseline characteristics and number of surveillance visits

	N (%)	Person-years	CRC cases	Incidence per 100 000 person-years (95% CI)	Univariable HR (95% CI)	P value	Multivariable HR (95% CI)	P value
Total	2719 (100)	22 961	84	366 (295 to 453)	–	–	–	–
Number of surveillance visits*	–	–	–	–	–	0.0019	–	0.0009
0	911 (33.5)	9243	44	476 (354 to 640)	1	–	1	–
1	695 (25.6)	7144	24	336 (225 to 501)	0.51 (0.30 to 0.85)	–	0.49 (0.29 to 0.82)	–
2	593 (21.8)	4018	10	249 (134 to 463)	0.32 (0.16 to 0.67)	–	0.30 (0.15 to 0.62)	–
≥3	520 (19.1)	2555	6	235 (105 to 523)	0.31 (0.12 to 0.78)	–	0.29 (0.11 to 0.73)	–
Sex	–	–	–	–	–	0.32	–	0.56
Women	799 (29.4)	6997	30	429 (300 to 613)	1	–	1	–
Men	1920 (70.6)	15 963	54	338 (259 to 442)	0.79 (0.51 to 1.24)	–	0.87 (0.56 to 1.37)	–
Age, years	–	–	–	–	–	0.0118	–	0.0828
<55	283 (10.4)	3191	6	188 (84 to 418)	1	–	1	–
55–64	750 (27.6)	7082	20	282 (182 to 438)	1.53 (0.61 to 3.81)	–	1.68 (0.67 to 4.19)	–
65–74	1065 (39.2)	8735	34	389 (278 to 545)	2.13 (0.89 to 5.09)	–	2.17 (0.91 to 5.19)	–
≥75	621 (22.8)	3953	24	607 (407 to 906)	3.42 (1.39 to 8.42)	–	2.79 (1.13 to 6.89)	–
Number of adenomas	–	–	–	–	–	0.49	–	0.38
3	1227 (45.1)	10 577	38	359 (261 to 494)	1	–	1	–
4	557 (20.5)	4704	13	276 (160 to 476)	0.77 (0.41 to 1.45)	–	0.81 (0.43 to 1.53)	–
5	454 (16.7)	3697	18	487 (307 to 773)	1.35 (0.77 to 2.36)	–	1.45 (0.83 to 2.54)	–
≥6	481 (17.7)	3983	15	377 (227 to 625)	1.05 (0.58 to 1.92)	–	1.24 (0.68 to 2.27)	–
Adenoma size, mm†	–	–	–	–	–	0.30	–	0.69
<10	264 (9.7)	2374	6	253 (114 to 562)	1	–	1	–
10–19	1344 (49.4)	11 361	36	317 (229 to 439)	1.25 (0.53 to 2.97)	–	1.08 (0.45 to 2.57)	–
≥20	1084 (39.9)	8951	41	458 (337 to 622)	1.82 (0.77 to 4.28)	–	1.41 (0.58 to 3.39)	–
Unknown	27 (1.0)	275	1	364 (51 to 2585)	1.41 (0.17 to 11.76)	–	1.22 (0.14 to 10.31)	–
Adenoma histology‡	–	–	–	–	–	0.19	–	0.48
Tubular	1038 (38.2)	8994	31	345 (242 to 490)	1	–	1	–
Tubulovillous	1293 (47.6)	10 701	36	336 (243 to 466)	0.99 (0.61 to 1.59)	–	0.89 (0.55 to 1.45)	–
Villous	328 (12.1)	2648	16	604 (370 to 986)	1.77 (0.97 to 3.23)	–	1.40 (0.75 to 2.61)	–
Unknown	60 (2.2)	619	1	162 (23 to 1147)	0.47 (0.06 to 3.41)	–	0.54 (0.07 to 4.09)	–
Adenoma dysplasia‡	–	–	–	–	–	0.0027	–	0.0009
Low-grade	2035 (74.8)	17 109	51	298 (227 to 392)	1	–	1	–
High-grade	616 (22.7)	5080	32	630 (445 to 891)	2.12 (1.36 to 3.30)	–	2.23 (1.43 to 3.47)	–
Unknown	68 (2.5)	772	1	130 (18 to 919)	0.44 (0.06 to 3.21)	–	0.35 (0.05 to 2.57)	–
Proximal polyps	–	–	–	–	–	0.96	–	0.47
No	663 (24.4)	5934	22	371 (244 to 563)	1	–	1	–
Yes	2056 (75.6)	17 027	62	364 (284 to 467)	0.99 (0.61 to 1.61)	–	1.21 (0.73 to 2.00)	–
Completeness of colonoscopy	–	–	–	–	–	0.0612	–	0.0438
Complete	2354 (86.6)	19 266	64	332 (260 to 424)	1	–	1	–
Incomplete	123 (4.5)	1009	7	694 (331 to 1456)	1.66 (1.00 to 2.76)	–	1.73 (1.04 to 2.89)	–
Unknown	242 (8.9)	2686	13	484 (281 to 833)		–		–
Bowel preparation quality	–	–	–	–	–	0.89	–	0.89
Excellent or good	1119 (41.2)	9788	35	358 (257 to 498)	1	–	1	–
Satisfactory	411 (15.1)	3106	12	386 (219 to 680)	1.08 (0.56 to 2.08)	–	1.03 (0.53 to 1.99)	–
Poor	143 (5.3)	980	5	510 (212 to 1226)	1.46 (0.57 to 3.72)	–	1.42 (0.55 to 3.64)	–
Unknown	1046 (38.5)	9086	32	352 (249 to 498)	0.99 (0.61 to 1.59)	–	0.95 (0.59 to 1.53)	–
Year of baseline visit	–	–	–	–	–	0.41	–	0.36
1984–1999	329 (12.1)	3948	10	253 (136 to 471)	1	–	1	–
2000–2004	874 (32.1)	8250	34	412 (294 to 577)	1.62 (0.78 to 3.38)	–	1.65 (0.78 to 3.47)	–
2005–2010	1516 (55.8)	10 762	40	372 (273 to 507)	1.48 (0.70 to 3.13)	–	1.65 (0.75 to 3.62)	–
Length of baseline visit	–	–	–	–	–	0.60	–	0.88
1 day	1184 (43.6)	10 106	33	327 (232 to 459)	1	–	1	–
2 days–3 months	562 (20.7)	4556	18	395 (249 to 627)	1.20 (0.68 to 2.14)	–	1.02 (0.57 to 1.82)	–
3–6 months	442 (16.3)	3738	12	321 (182 to 565)	0.98 (0.51 to 1.89)	–	0.89 (0.46 to 1.74)	–
≥6 months	531 (19.5)	4561	21	460 (300 to 706)	1.42 (0.82 to 2.45)	–	1.19 (0.67 to 2.10)	–

The final multivariable model contained number of surveillance visits, completeness of colonoscopy and adenoma dysplasia. For these variables, the multivariable HRs were from the final multivariable model and the p values were for inclusion of the variable in the model, calculated with the likelihood ratio test. For the remaining variables, the multivariable HRs were for if the variable was added as an additional variable to the final multivariable model.

*The number of surveillance visits was included as a time-varying covariate; patients who attended any surveillance contributed person-years to more than one category of number of surveillance visits.

†Adenoma size was defined according to the largest adenoma seen at baseline.

‡Adenoma histology and dysplasia were defined according to the worst histology (greatest degree of villousness) and worst dysplasia (highest grade) seen at baseline, respectively.

There were no significant interactions between number of surveillance visits and age or sex (all p values ≥0.05). Each risk group was then divided into lower-risk and higher-risk subgroups using the identified baseline risk factors.

### Low-risk group

The higher-risk subgroup of low-risk patients comprised those with incomplete colonoscopies, colonoscopies of unknown completeness, tubulovillous or villous adenomas, or proximal polyps at baseline (n=9166, 64%); lower-risk patients had none of these (n=5235, 36%) (table 4). Higher-risk patients were older, more likely to have had a baseline visit before 2005, and had more surveillance than lower-risk patients (online supplementary table 2). Surveillance was associated with lower CRC incidence in the higher-risk but not the lower-risk subgroup; however, estimates in the lower-risk subgroup were imprecise owing to few CRCs (table 4).

**Table 4:** Incidence of colorectal cancer (CRC) and unadjusted effect of surveillance on CRC incidence by number of surveillance visits

	n (%)	Person-years	CRC cases	Incidence per 100 000 person-years (95% CI)	Effect of surveillance*	
					Univariable HR (95% CI)	P value
**Low-risk group**						
Whole risk group	–	–	–	–	–	<0.0001
0 visits	7207 (50.1)	84 591	143	169 (143 to 199)	1	–
1 visit	3959 (27.5)	34 507	41	119 (87 to 161)	0.55 (0.39 to 0.79)	–
≥2 visits	3235 (22.5)	19 805	11	56 (31 to 100)	0.23 (0.12 to 0.44)	–
Total	14 401 (100)	138 903	195	140 (122 to 162)	–	–
Lower-risk subgroup†	–	–	–	–	–	0.15
0 visits	2804 (53.6)	32 903	31	94 (66 to 134)	1	–
1 visit	1432 (27.4)	11 410	8	70 (35 to 140)	0.54 (0.25 to 1.20)	–
≥2 visits	999 (19.1)	5472	3	55 (18 to 170)	0.42 (0.12 to 1.48)	–
Total	5235 (36.4)	49 785	42	84 (62 to 114)	–	–
Higher-risk subgroup†	–	–	–	–	–	<0.0001
0 visits	4403 (48.0)	51 688	112	217 (180 to 261)	1	–
1 visit	2527 (27.6)	23 097	33	143 (102 to 201)	0.52 (0.35 to 0.78)	–
≥2 visits	2236 (24.4)	14 332	8	56 (28 to 112)	0.18 (0.08 to 0.37)	–
Total	9166 (63.6)	89 118	153	172 (147 to 201)	–	–
**Intermediate-risk group**						
Whole risk group	–	–	–	–	–	0.0001
0 visits	4683 (39.5)	53 927	135	250 (211 to 296)	1	–
1 visit	3343 (28.2)	33 284	62	186 (145 to 239)	0.58 (0.42 to 0.79)	–
≥2 visits	3826 (32.3)	24 059	49	204 (154 to 269)	0.50 (0.34 to 0.73)	–
Total	11 852 (100)	111 270	246	221 (195 to 251)	–	–
Lower-risk subgroup‡	–	–	–	–	–	0.30
0 visits	1932 (40.8)	23 237	33	142 (101 to 200)	1	–
1 visit	1387 (29.3)	13 151	16	122 (75 to 199)	0.66 (0.35 to 1.23)	–
≥2 visits	1419 (30.0)	8082	13	161 (93 to 277)	0.63 (0.31 to 1.29)	–
Total	4738 (40.0)	44 470	62	139 (109 to 179)	–	–
Higher-risk subgroup‡	–	–	–	–	–	0.0001
0 visits	2751 (38.7)	30 690	102	332 (274 to 404)	1	–
1 visit	1956 (27.5)	20 133	46	228 (171 to 305)	0.53 (0.37 to 0.76)	–
≥2 visits	2407 (33.8)	15 977	36	225 (163 to 312)	0.42 (0.27 to 0.65)	–
Total	7114 (60.0)	66 800	184	275 (238 to 318)	–	–
**High-risk group**						
Whole risk group	–	–	–	–	–	0.0006
0 visits	911 (33.5)	9243	44	476 (354 to 640)	1	–
1 visit	695 (25.6)	7144	24	336 (225 to 501)	0.51 (0.30 to 0.85)	–
≥2 visits	1113 (40.9)	6574	16	243 (149 to 397)	0.32 (0.17 to 0.60)	–
Total	2719 (100)	22 961	84	366 (295 to 453)	–	–
Lower-risk subgroup§	–	–	–	–	–	0.26
0 visits	606 (33.4)	6226	17	273 (170 to 439)	1	–
1 visit	474 (26.1)	4766	12	252 (143 to 443)	0.66 (0.31 to 1.41)	–
≥2 visits	737 (40.6)	4039	9	223 (116 to 428)	0.49 (0.20 to 1.18)	–
Total	1817 (66.8)	15 032	38	253 (184 to 347)	–	–
Higher-risk subgroup§	–	–	–	–	–	0.0006
0 visits	305 (33.8)	3017	27	895 (614 to 1305)	1	–
1 visit	221 (24.5)	2378	12	505 (287 to 889)	0.41 (0.20 to 0.82)	–
≥2 visits	376 (41.7)	2535	7	276 (132 to 579)	0.21 (0.08 to 0.51)	–
Total	902 (33.2)	7929	46	580 (435 to 775)	–	–

P values calculated with the likelihood ratio test.

*The number of surveillance visits was included as a time-varying covariate; patients who attended any surveillance contributed person-years to more than one category of number of surveillance visits.

†The higher-risk subgroup included patients with an incomplete colonoscopy or colonoscopy of unknown completeness, a tubulovillous or villous adenoma, or proximal polyps at baseline; the lower-risk subgroup included patients with none of these factors.

‡The higher-risk subgroup included patients with an incomplete colonoscopy or colonoscopy of unknown completeness, an adenoma with high-grade dysplasia or proximal polyps at baseline; the lower-risk subgroup included patients with none of these factors.

§The higher-risk subgroup included patients with an incomplete colonoscopy or colonoscopy of unknown completeness or an adenoma with high-grade dysplasia at baseline; the lower-risk subgroup included patients with none of these factors.

**Figure 2:**
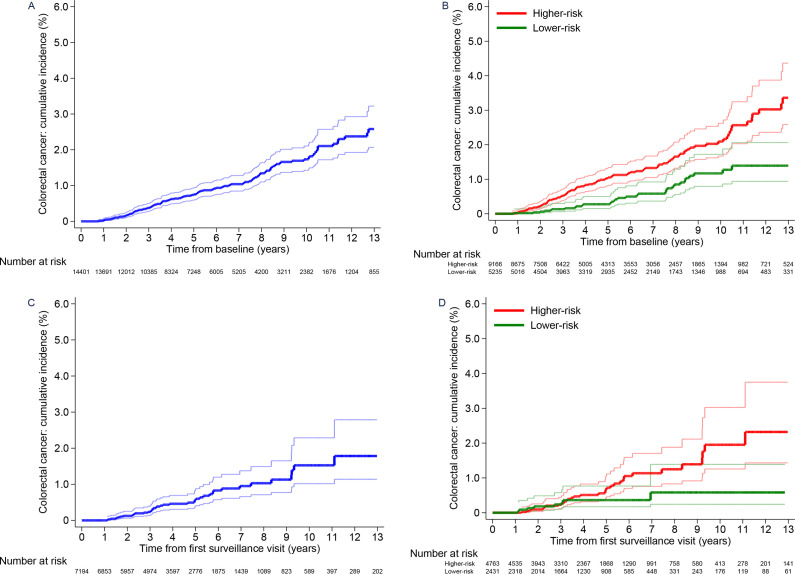
Cumulative colorectal cancer incidence after baseline in the low-risk group.Cumulative colorectal cancer incidence with no surveillance (censoring at first surveillance) for the whole low-risk group (A) and the lower-risk and higher-risk subgroups (B). Cumulative colorectal cancer incidence after a single surveillance visit (censoring at second surveillance) for the whole low-risk group (C) and the lower-risk and higher-risk subgroups (D). 95% CIs are shown for each curve. The higher-risk subgroup included patients with an incomplete colonoscopy or colonoscopy of unknown completeness, a tubulovillous or villous adenoma, or proximal polyps at baseline; the lower-risk subgroup included patients with none of these factors.

Without surveillance, cumulative CRC incidence at 10 years was 1.7% (95% CI 1.4 to 2.1) in the whole low-risk group, differing significantly between the lower-risk (1.2%, 95% CI 0.8 to 1.7) and higher-risk subgroups (2.1%, 95% CI 1.7 to 2.6) (table 5; figure 2). Compared with the general population, CRC incidence was similar in the whole low-risk group (SIR 0.86, 95% CI 0.73 to 1.02) and higher-risk subgroup (SIR 1.07, 95% CI 0.88 to 1.28), but was lower in the lower-risk subgroup (SIR 0.51, 95% CI 0.35 to 0.73) (table 5). After first surveillance, cumulative CRC incidence was lower (table 5; figure 2) and incidence in both subgroups was below that in the general population (table 5).

**Table 5:** Cumulative colorectal cancer (CRC) incidence and age-standardised and sex-standardised incidence ratios

	n (%)	Person-years	CRC cases	Incidence per 100 000 person-years (95% CI)	Follow-up						P value	Standardisation	
					3 years		5 years		10 years				
					CRC cases	Cumulative incidence (95% CI)*	CRC cases	Cumulative incidence (95% CI)*	CRC cases	Cumulative incidence (95% CI)*		Expected CRCs†	SIR (95% CI)
**Low-risk group**	–	–	–	–	–	–	–	–	–	–	–	–	–
After baseline (no surveillance, censored at first surveillance)	–	–	–	–	–	–	–	–	–	–	<0.0001	–	–
Whole risk group	14 401 (100)	84 591	143	169 (143 to 199)	45	0.4% (0.3 to 0.5)	77	0.7% (0.6 to 0.9)	124	1.7% (1.4 to 2.1)	–	165	0.86 (0.73 to 1.02)
Lower-risk subgroup‡	5235 (36)	32 903	31	94 (66 to 134)	6	0.1% (0.1 to 0.3)	11	0.3% (0.2 to 0.5)	29	1.2% (0.8 to 1.7)	–	60	0.51 (0.35 to 0.73)
Higher-risk subgroup‡	9166 (64)	51 688	112	217 (180 to 261)	39	0.5% (0.4 to 0.7)	66	1.0% (0.8 to 1.3)	95	2.1% (1.7 to 2.6)	–	105	1.07 (0.88 to 1.28)
After first surveillance (single surveillance visit, censored at second surveillance)	–	–	–	–	–	–	–	–	–	–	0.0691	–	–
Whole risk group	7194 (100)	34 507	41	119 (87 to 161)	14	0.2% (0.1 to 0.4)	27	0.6% (0.4 to 0.8)	40	1.5% (1.0 to 2.3)	–	69	0.60 (0.43 to 0.81)
Lower-risk subgroup‡	2431 (34)	11 410	8	70 (35 to 140)	5	0.2% (0.1 to 0.6)	7	0.4% (0.2 to 0.8)	8	0.6% (0.2 to 1.4)	–	21	0.38 (0.16 to 0.74)
Higher-risk subgroup‡	4763 (66)	23 097	33	143 (102 to 201)	9	0.2% (0.1 to 0.4)	20	0.7% (0.4 to 1.0)	32	2.0% (1.3 to 3.0)	–	47	0.70 (0.48 to 0.98)
After second surveillance (two or more surveillance visits, censored at end of follow-up)	–	–	–	–	–	–	–	–	–	–	0.90	–	–
Whole risk group	3235 (100)	19 805	11	56 (31 to 100)	1	0.04% (0.01 to 0.3)	4	0.2% (0.1 to 0.5)	8	0.6% (0.3 to 1.3)	–	42	0.26 (0.13 to 0.47)
Lower-risk subgroup‡	999 (31)	5472	3	55 (18 to 170)	0	–	0	–	2	0.7% (0.2 to 3.0)	–	10	0.29 (0.06 to 0.84)
Higher-risk subgroup‡	2236 (69)	14 332	8	56 (28 to 112)	1	0.1% (0.01 to 0.4)	4	0.3% (0.1 to 0.7)	6	0.6% (0.2 to 1.4)	–	32	0.25 (0.11 to 0.50)
**Intermediate-risk group**	–	–	–	–	–	–	–	–	–	–	–	–	–
After baseline (no surveillance, censored at first surveillance)	–	–	–	–	–	–	–	–	–	–	<0.0001	–	–
Whole risk group	11 852 (100)	53 927	135	250 (211 to 296)	57	0.6% (0.5 to 0.8)	88	1.3% (1.0 to 1.6)	121	2.6% (2.1 to 3.3)	–	117	1.16 (0.97 to 1.37)
Lower-risk subgroup§	4738 (40)	23 237	33	142 (101 to 200)	13	0.4% (0.2 to 0.6)	20	0.7% (0.4 to 1.1)	27	1.3% (0.8 to 2.1)	–	47	0.70 (0.48 to 0.99)
Higher-risk subgroup§	7114 (60)	30 690	102	332 (274 to 404)	44	0.8% (0.6 to 1.1)	68	1.7% (1.3 to 2.2)	94	3.7% (2.9 to 4.7)	–	70	1.46 (1.19 to 1.78)
After first surveillance (single surveillance visit, censored at second surveillance)	–	–	–	–	–	–	–	–	–	–	0.0292	–	–
Whole risk group	7169 (100)	33 284	62	186 (145 to 239)	17	0.3% (0.2 to 0.5)	34	0.8% (0.6 to 1.2)	57	2.6% (1.9 to 3.6)	–	73	0.85 (0.65 to 1.08)
Lower-risk subgroup§	2806 (39)	13 151	16	122 (75 to 199)	3	0.1% (0.04 to 0.4)	8	0.5% (0.2 to 1.0)	15	1.9% (1.0 to 3.4)	–	27	0.59 (0.34 to 0.96)
Higher-risk subgroup§	4363 (61)	20 133	46	228 (171 to 305)	14	0.4% (0.2 to 0.7)	26	1.0% (0.7 to 1.5)	42	3.1% (2.2 to 4.5)	–	46	1.00 (0.73 to 1.33)
After second surveillance (two or more surveillance visits, censored at end of follow-up)	–	–	–	–	–	–	–	–	–	–	0.39	–	–
Whole risk group	3826 (100)	24 059	49	204 (154 to 269)	10	0.3% (0.2 to 0.6)	19	0.7% (0.4 to 1.1)	36	2.0% (1.4 to 2.9)	–	56	0.87 (0.65 to 1.16)
Lower-risk subgroup§	1419 (37)	8082	13	161 (93 to 277)	3	0.3% (0.1 to 0.8)	6	0.6% (0.3 to 1.5)	10	1.7% (0.8 to 3.3)	–	18	0.72 (0.38 to 1.22)
Higher-risk subgroup§	2407 (63)	15 977	36	225 (163 to 312)	7	0.3% (0.2 to 0.7)	13	0.7% (0.4 to 1.2)	26	2.2% (1.4 to 3.3)	–	38	0.95 (0.67 to 1.31)
**High-risk group**	–	–	–	–	–	–	–	–	–	–	–	–	–
After baseline (no surveillance, censored at first surveillance)	–	–	–	–	–	–	–	–	–	–	0.0001	–	–
Whole risk group	2719 (100)	9243	44	476 (354 to 640)	17	1.0% (0.6 to 1.7)	32	3.1% (2.1 to 4.4)	41	5.7% (4.0 to 8.3)	–	23	1.91 (1.39 to 2.56)
Lower-risk subgroup¶	1817 (67)	6226	17	273 (170 to 439)	7	0.6% (0.3 to 1.4)	13	1.9% (1.0 to 3.4)	17	3.8% (2.1 to 6.8)	–	15	1.10 (0.64 to 1.76)
Higher-risk subgroup¶	902 (33)	3017	27	895 (614 to 1305)	10	1.9% (1.0 to 3.6)	19	5.6% (3.5 to 9.0)	24	9.9% (6.2 to 15.7)	–	8	3.55 (2.34 to 5.17)
After first surveillance (single surveillance visit, censored at second surveillance)	–	–	–	–	–	–	–	–	–	–	0.0864	–	–
Whole risk group	1808 (100)	7144	24	336 (225 to 501)	9	0.6% (0.3 to 1.2)	16	1.8% (1.1 to 3.1)	23	5.6% (3.1 to 9.8)	–	18	1.34 (0.86 to 1.99)
Lower-risk subgroup¶	1211 (67)	4766	12	252 (143 to 443)	5	0.5% (0.2 to 1.3)	8	1.3% (0.6 to 2.9)	11	4.4% (1.8 to 10.6)	–	12	1.01 (0.52 to 1.76)
Higher-risk subgroup¶	597 (33)	2378	12	505 (287 to 889)	4	0.9% (0.3 to 2.3)	8	2.9% (1.4 to 6.1)	12	7.8% (3.8 to 15.4)	–	6	1.97 (1.02 to 3.44)
After second surveillance (two or more surveillance visits, censored at end of follow-up)	–	–	–	–	–	–	–	–	–	–	0.74	–	–
Whole risk group	1113 (100)	6574	16	243 (149 to 397)	3	0.3% (0.1 to 1.0)	9	1.2% (0.6 to 2.3)	15	2.6% (1.5 to 4.4)	–	18	0.91 (0.52 to 1.47)
Lower-risk subgroup¶	737 (66)	4039	9	223 (116 to 428)	1	0.2% (0.02 to 1.2)	6	1.3% (0.6 to 2.8)	9	2.4% (1.2 to 4.7)	–	11	0.83 (0.38 to 1.58)
Higher-risk subgroup¶	376 (34)	2535	7	276 (132 to 579)	2	0.6% (0.2 to 2.4)	3	1.0% (0.3 to 3.1)	6	2.7% (1.2 to 6.2)	–	7	1.02 (0.41 to 2.09)

P values calculated with the log-rank test to compare incidence in the lower-risk and higher-risk subgroups of each risk group.

*One minus the Kaplan-Meier estimator of the survival function was used to estimate the cumulative incidence of colorectal cancer.

†Expected numbers of colorectal cancers were calculated by multiplying the sex and 5-year age-group-specific observed person-years by the corresponding incidence rates in the general population of England in 2007.

‡The higher-risk subgroup included patients with an incomplete colonoscopy or colonoscopy of unknown completeness, a tubulovillous or villous adenoma, or proximal polyps at baseline; the lower-risk subgroup included patients with none of these factors.

§The higher-risk subgroup included patients with an incomplete colonoscopy or colonoscopy of unknown completeness, an adenoma with high-grade dysplasia or proximal polyps at baseline; the lower-risk subgroup included patients with none of these factors.

¶The higher-risk subgroup included patients with an incomplete colonoscopy or colonoscopy of unknown completeness or an adenoma with high-grade dysplasia at baseline; the lower-risk subgroup included patients with none of these factors.

SIR, standardised incidence ratio.

### Intermediate-risk group

The higher-risk subgroup of intermediate-risk patients comprised those with incomplete colonoscopies, colonoscopies of unknown completeness, adenomas with high-grade dysplasia or proximal polyps at baseline (n=7114, 60%); lower-risk patients had none of these (n=4738, 40%) (table 4). Higher-risk patients were older, more likely to have had a baseline visit before 2005, and had more surveillance than lower-risk patients (online supplementary table 2). Surveillance was associated with reduced CRC incidence in the higher-risk but not the lower-risk subgroup, although estimates in the lower-risk subgroup were imprecise (table 4).

**Figure 3:**
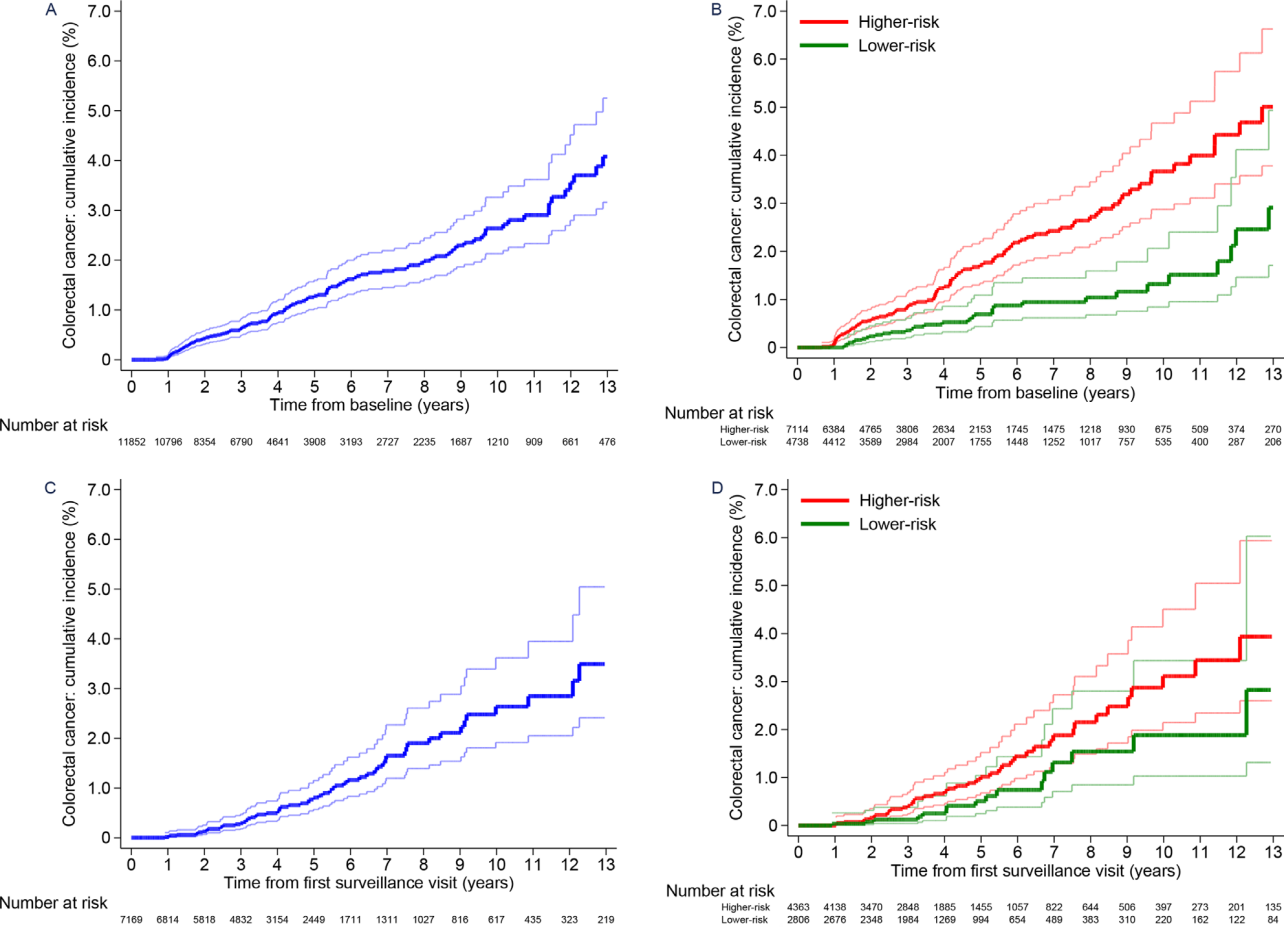
Cumulative colorectal cancer incidence after baseline in the intermediate-risk group.Cumulative colorectal cancer incidence with no surveillance (censoring at first surveillance) for the whole intermediate-risk group (A) and the lower-risk and higher-risk subgroups (B). Cumulative colorectal cancer incidence after a single surveillance visit (censoring at second surveillance) for the whole intermediate-risk group (C) and the lower-risk and higher-risk subgroups (D). 95% CIs are shown for each curve. The higher-risk subgroup included patients with an incomplete colonoscopy or colonoscopy of unknown completeness, an adenoma with high-grade dysplasia or proximal polyps at baseline; the lower-risk subgroup included patients with none of these factors.

Without surveillance, cumulative CRC incidence at 10 years was 2.6% (95% CI 2.1 to 3.3) in the whole intermediate-risk group, differing significantly between the lower-risk (1.3%, 95% CI 0.8 to 2.1) and higher-risk (3.7%, 95% CI 2.9 to 4.7) subgroups (table 5; figure 3). Compared with the general population, CRC incidence was similar in the whole intermediate-risk group (SIR 1.16, 95% CI 0.97 to 1.37), lower in the lower-risk subgroup (SIR 0.70, 95% CI 0.48 to 0.99) and higher in the higher-risk subgroup (SIR 1.46, 95% CI 1.19 to 1.78) (table 5).

After first surveillance, cumulative CRC incidence still differed between the risk subgroups (table 5; figure 3), although incidence in the higher-risk subgroup was now similar to that in the general population (SIR 1.00, 95% CI 0.73 to 1.33) and was lower in the lower-risk subgroup (SIR 0.59, 95% CI 0.34 to 0.96) (table 5).

When we additionally included poor bowel preparation and adenomas ≥20 mm in the classification of higher risk, the proportion of patients classed as higher risk increased to 74%. Incidence rates and effects of surveillance on incidence remained similar (data not shown).

### High-risk group

The higher-risk subgroup of high-risk patients included those with incomplete colonoscopies, colonoscopies of unknown completeness or adenomas with high-grade dysplasia at baseline (n=902, 33%); lower-risk patients had none of these (n=1817, 67%) (table 4). The subgroups were similar regarding sex, age, year of baseline visit and number of surveillance visits (online supplementary table 2). Surveillance was associated with reduced CRC incidence in the higher-risk but not the lower-risk subgroup, although estimates in the lower-risk subgroup were imprecise (table 4).

**Figure 4:**
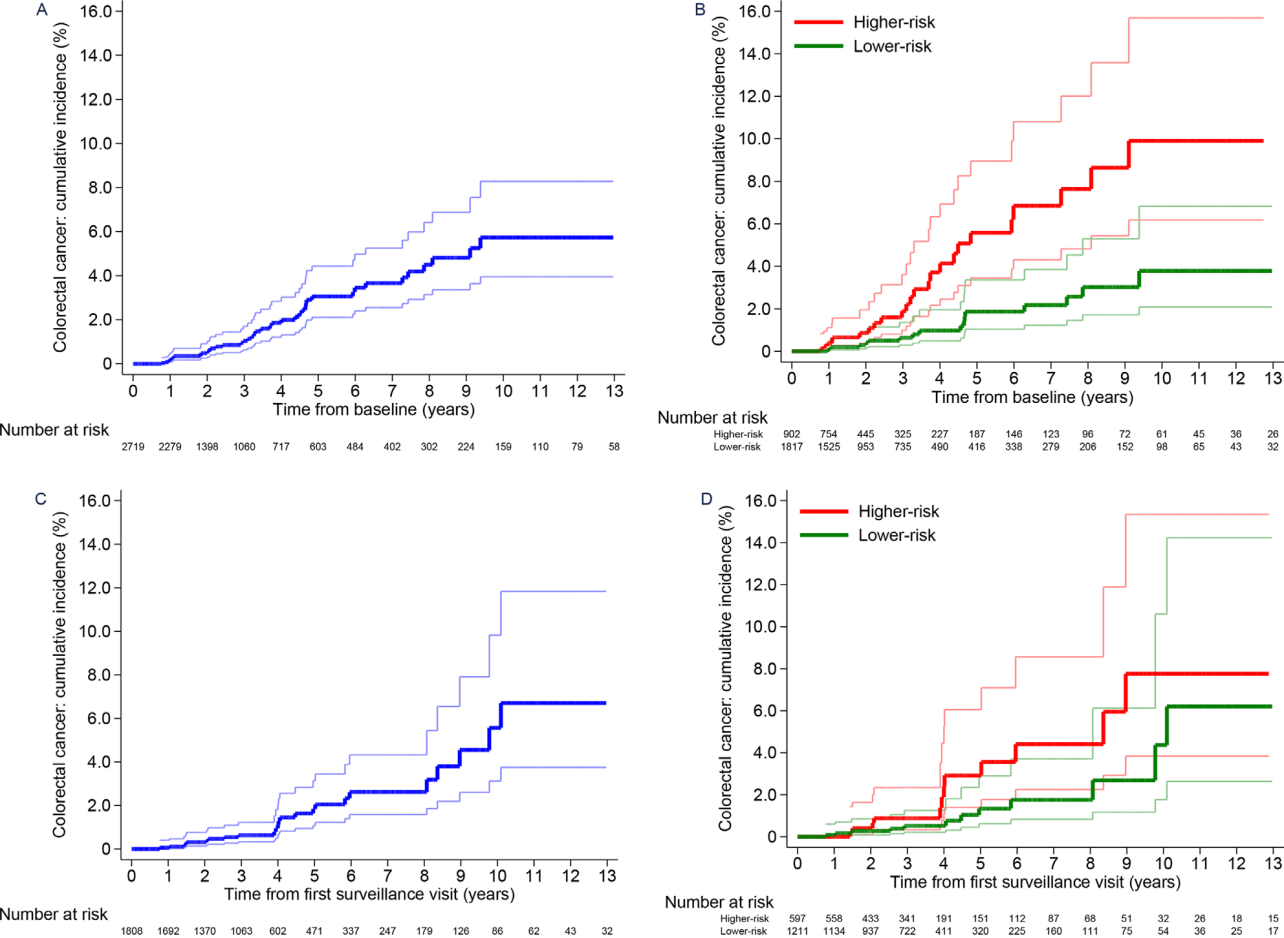
Cumulative colorectal cancer incidence after baseline in the high-risk group.Cumulative colorectal cancer incidence with no surveillance (censoring at first surveillance) for the whole high-risk group (A) and the lower-risk and higher-risk subgroups (B). Cumulative colorectal cancer incidence after a single surveillance visit (censoring at second surveillance) for the whole high-risk group (C) and the lower-risk and higher-risk subgroups (D). 95% CIs are shown for each curve. The higher-risk subgroup included patients with an incomplete colonoscopy or colonoscopy of unknown completeness or an adenoma with high-grade dysplasia at baseline; the lower-risk subgroup included patients with none of these factors.

Without surveillance, cumulative CRC incidence at 10 years was 5.7% (95% CI 4.0 to 8.3) in the whole high-risk group, differing significantly between the lower-risk (3.8%, 95% CI 2.1 to 6.8) and higher-risk subgroups (9.9%, 95% CI 6.2 to 15.7) (table 5; figure 4). Compared with the general population, CRC incidence was higher in the whole high-risk group (SIR 1.91, 95% CI 1.39 to 2.56) and higher-risk subgroup (SIR 3.55, 95% CI 2.34 to 5.17), but not significantly different in the lower-risk subgroup (SIR 1.10, 95% CI 0.64 to 1.76) (table 5).

After first surveillance, cumulative CRC incidence at 10 years was 5.6% (95% CI 3.1 to 9.8) in the whole high-risk group, 4.4% (95% CI 1.8 to 10.6) in the lower-risk subgroup and 7.8% (95% CI 3.8 to 15.4) in the higher-risk subgroup (table 5; figure 4). Compared with the general population, CRC incidence was not significantly different in the whole high-risk group (SIR 1.34, 95% CI 0.86 to 1.99) or lower-risk subgroup (SIR 1.01, 95% CI 0.52 to 1.76), but remained higher in the higher-risk subgroup (SIR 1.97, 95% CI 1.02 to 3.44). After a second surveillance visit, CRC incidence was no longer higher in the higher-risk subgroup than in the general population (table 5).

In the main analysis, we excluded CRCs assumed to have arisen from incompletely resected baseline lesions; those found in the same/adjacent colonic segment to a baseline adenoma ≥15 mm that was seen at least twice within 5 years preceding cancer diagnosis (intermediate-risk group, n=38; high-risk group, n=12). In sensitivity analyses, we additionally excluded CRCs that satisfied only some of these criteria, but that we deemed likely to have arisen from incompletely resected lesions (low-risk group, n=6; intermediate-risk group, n=29; high-risk group, n=7). This negligibly affected the results (data not shown). Excluding patients without a complete baseline colonoscopy (low-risk group, n=2682; intermediate-risk group, n=2885; high-risk group, n=365) had little impact (online supplementary tables 3–7), although high-grade dysplasia was no longer significant in intermediate-risk patients (online supplementary table 4).

## Discussion

This is the largest study examining long-term CRC incidence following adenoma removal and the effects of surveillance on CRC incidence. We obtained data from 17 hospitals on 28 972 patients who underwent baseline colonoscopy and polypectomy and were followed for a median of 9.3 years. Stratifying the cohort into low-risk (50%), intermediate-risk (41%) and high-risk (9%) groups according to the 2002 UK surveillance guidelines,^3^ we identified heterogeneity in CRC incidence and in the effects of surveillance on CRC incidence among each risk group.

Our analyses showed that patients in the low-risk group were indeed at low risk of CRC. Even among the two-thirds of the group at higher CRC risk than the rest owing to an incomplete colonoscopy, colonoscopy of unknown completeness, tubulovillous or villous adenoma, or proximal polyps at baseline, CRC incidence was similar to that in the general population, without any surveillance. Among the remaining one-third, CRC incidence without surveillance was lower than in the general population. In a resource-constrained setting, it is important to consider the opportunity costs of performing surveillance in a particular patient group; we think that patients remaining at increased CRC risk following a high-quality baseline colonoscopy, as compared with the general population, should be prioritised. Given this, and considering the risks of colonoscopy, we think that patients classified as low-risk do not require surveillance and they could instead be managed by screening.

Our results corroborated our previous finding that surveillance is warranted for most but probably not all intermediate-risk patients.^13^ Among intermediate-risk patients with incomplete colonoscopies, colonoscopies of unknown completeness, adenomas with high-grade dysplasia or proximal polyps at baseline (60% of the risk group), CRC incidence without surveillance was higher than in the general population and a single surveillance visit conferred substantial protection against CRC. Among patients without these characteristics, CRC incidence was lower than in the general population after baseline colonoscopy, indicating that surveillance is not necessary.

Incidence of CRC was high in the high-risk group; without surveillance, rates were double that in the general population. Cumulative incidence at 10 years was 6% both without surveillance and with one surveillance visit, falling to 3% with two visits. High-risk patients might therefore benefit from attending two surveillance visits, although studies are needed to define the optimum interval between first and second visits. When we stratified the high-risk group into subgroups, estimates were too imprecise to draw clear conclusions.

Our findings suggest that surveillance is warranted for high-risk patients (n=2719) and the higher-risk subgroup of intermediate-risk patients (n=7114) (34% of our cohort), but not for the lower-risk subgroup of intermediate-risk patients (n=4738) or low-risk patients (n=14 401) (66% of our cohort), who could instead be managed by screening. In the BCSP in England, surveillance is recommended for intermediate-risk and high-risk patients only.^23^ In this setting, numbers of surveillance colonoscopies could be reduced by a third if the lower-risk subgroup of intermediate-risk patients forewent surveillance.

Patients returning to the BCSP would be screened biennially with the faecal immunochemical test (FIT), which replaced the faecal occult blood test in June 2019.^24^ Although FIT was introduced with a relatively high positivity threshold of 120 µg haemoglobin per gram of faeces, the threshold may be lowered over time if endoscopy capacity increases, which would improve FIT sensitivity for adenomas and early CRCs.^25^ It is important that patients returning to screening are reminded to see their general practitioner if lower gastrointestinal symptoms occur.

Several baseline characteristics were repeatedly predictive of CRC, including older age, incomplete colonoscopies, adenomas with high-grade dysplasia and proximal polyps. This aligns with our previous study of intermediate-risk patients,^13 16^ and other studies describing these as risk factors for incident advanced neoplasia.^26 27^ These findings reinforce the importance of a thorough baseline colonoscopy with complete resection of detected lesions. Incomplete resection might be implicated in the elevated risk among patients with high-grade dysplasia or proximal polyps, as advanced and proximal polyps have been associated with greater risks of incomplete resection.^28^ Some proximal polyps in our study may have been serrated lesions which are often proximally located, flat, and difficult to see and remove.^29^ Unfortunately, serrated lesions were not consistently classified in the era of our data.

Half of low-risk patients, 60% of intermediate-risk patients and 66% of high-risk patients attended surveillance. Non-attenders were older than attenders, more likely to have had an incomplete baseline colonoscopy or poor bowel preparation, and in the intermediate-risk group were more likely to be female, consistent with the literature.^13 16 30^ That 30%–40% of intermediate-risk and high-risk patients had no surveillance suggests some underuse of surveillance colonoscopy. Unfortunately, we had no information on why patients did not attend surveillance, but reasons may have included patient comorbidities, objections to colonoscopy or process errors.

Among low-risk patients, first surveillance occurred after a median of 3.2 years, earlier than recommended.^3^ This has been observed elsewhere.^31 32^ Possible explanations include slow adoption of guidelines and concern about postcolonoscopy CRCs. There was greater adherence to recommended surveillance intervals for intermediate-risk and high-risk patients.

Besides the present study and our previous study of intermediate-risk patients,^13 16^ only one other study has compared CRC risk following adenoma removal with that in the general population in the absence and presence of surveillance.^17^ This study included 5779 patients who underwent baseline colonoscopy from 1990 to 1999. Among patients with an AA (adenoma ≥10 mm, with high-grade dysplasia or villous histology) at baseline, CRC risk without surveillance was four times that in the general population and surveillance substantially reduced this risk. By contrast, among patients with non-AAs, CRC risk without surveillance was similar to in the general population and surveillance did not affect CRC risk. The study was limited, however, by the small sample size and age of the data.

Strengths of the present study include the large, high-quality data set, comprising detailed data from 17 hospitals on baseline and surveillance colonoscopies. The hospitals included general and teaching hospitals located throughout the UK. Few data were missing and follow-up was complete for nearly all patients. Most baseline colonoscopies were performed after the introduction of colonoscopy quality initiatives in 2001.^11^ Nevertheless, 20% of patients did not have a complete baseline colonoscopy. Exclusion of these patients had little impact, however, indicating that the findings are applicable in the modern era of high-quality colonoscopy.

Limitations include the observational design, meaning we cannot assume that surveillance caused the reductions in CRC incidence. However, we adjusted for potential confounders and still saw a large effect of surveillance on incidence. Use of routine data means that misclassification may have occurred; however, this would likely be non-differential, producing underestimations of effects. More patients attending surveillance were missing baseline data than non-attenders, particularly for colonoscopy completeness and bowel preparation quality, which is a potential source of bias. Some follow-up colonoscopies may have been for symptoms rather than surveillance. Additionally, as patients were stratified into risk groups by baseline adenoma size and number, we could not interpret the individual effects of these characteristics. Finally, although the follow-up period was long, the full benefit of surveillance on CRC incidence may not manifest until after 10 years.

## Conclusion

A large proportion of patients with adenomas do not remain at increased CRC risk following a complete baseline colonoscopy and polypectomy, compared with the general population. In our cohort, this was true for low-risk patients, and intermediate-risk patients without high-grade dysplasia or proximal polyps. Surveillance is probably not necessary for these patients and routine screening would suffice, although patients should be reminded to contact their general practitioner if lower gastrointestinal symptoms occur. Conversely, surveillance is warranted for high-risk patients, and intermediate-risk patients without a complete baseline colonoscopy or with high-grade dysplasia or proximal polyps, whose risk was higher than in the general population before surveillance. Incorporating these findings into guidelines could reduce surveillance colonoscopies by a third, while ensuring that patients at increased risk are protected.
