# Supplementary material for: Long-term colorectal cancer incidence after adenoma removal and the effects of surveillance on incidence: a multicentre, retrospective, cohort study
Source: Gut. 2020 Jan 17;69(9):1645–58. doi: 10.1136/gutjnl-2019-320036 (PMC7456728; doi:10.1136/gutjnl-2019-320036)
Supplement: Supplementary data [file gutjnl-2019-320036supp001.pdf]

BMJ Publishing Group Limited (BMJ) disclaims all liability and responsibility arising from any reliance placed on this supplemental material which has been supplied by the author(s)

Supplementary Table 1: Baseline patient, procedural, and polyp characteristics by surveillance visit attendance

	Low-risk group (n=14 401)					Intermediate-risk group (n=11 852)					High-risk group (n=2719)				
	Patients with ≥1 surveillance visits		Patients with no surveillance		p-value	Patients with ≥1 surveillance visits		Patients with no surveillance		p-value	Patients with ≥1 surveillance visits		Patients with no surveillance		p-value
	n	(%)	n	(%)		n	(%)	n	(%)		n	(%)	n	(%)	
Total	7194	(50.0)	7207	(50.0)	..	7169	(60.5)	4683	(39.5)	..	1808	(66.5)	911	(33.5)	..
Sex	..	..	..	..	0.32	..	..	..	..	0.023	..	..	..	..	0.46
Women	3218	(44.7)	3164	(43.9)	..	3128	(43.6)	2143	(45.8)	..	523	(28.9)	276	(30.3)	..
Men	3976	(55.3)	4043	(56.1)	..	4041	(56.4)	2540	(54.2)	..	1285	(71.1)	635	(69.7)	..
Age, years	..	..	..	..	<0.0001	..	..	..	..	<0.0001	..	..	..	..	<0.0001
<55	2147	(29.8)	1422	(19.7)	..	1537	(21.4)	560	(12.0)	..	221	(12.2)	62	(6.8)	..
55-64	2339	(32.5)	1652	(22.9)	..	2278	(31.8)	880	(18.8)	..	602	(33.3)	148	(16.2)	..
65-74	2007	(27.9)	2251	(31.2)	..	2460	(34.3)	1455	(31.1)	..	719	(39.8)	346	(38.0)	..
≥75	701	(9.7)	1882	(26.1)	..	894	(12.5)	1788	(38.2)	..	266	(14.7)	355	(39.0)	..
Number of adenomas	..	..	..	..	<0.0001	..	..	..	..	0.041	..	..	..	..	0.0015
1	5753	(80.0)	6009	(83.4)	..	4701	(65.6)	3092	(66.0)	..	0	(0)	0	(0)	..
2	1441	(20.0)	1198	(16.6)	..	1863	(26.0)	1190	(25.4)	..	0	(0)	0	(0)	..
3	0	(0)	0	(0)	..	416	(5.8)	308	(6.6)	..	788	(43.6)	439	(48.2)	..
4	0	(0)	0	(0)	..	189	(2.6)	93	(2.0)	..	367	(20.3)	190	(20.9)	..
5	0	(0)	0	(0)	..	0	(0)	0	(0)	..	297	(16.4)	157	(17.2)	..
≥6	0	(0)	0	(0)	..	0	(0)	0	(0)	..	356	(19.7)	125	(13.7)	..
Adenoma size, mm†	..	..	..	..	..	..	..	..	..	0.0003	..	..	..	..	0.13
<10	7194	(100)	7207	(100)	..	605	(8.4)	401	(8.6)	..	189	(10.5)	75	(8.2)	..
10-19	0	(0)	0	(0)	..	4018	(56.0)	2784	(59.4)	..	870	(48.1)	474	(52.0)	..
≥20	0	(0)	0	(0)	..	2546	(35.5)	1498	(32.0)	..	732	(40.5)	352	(38.6)	..
Unknown	0	(0)	0	(0)	..	0	(0)	0	(0)	..	17	(0.9)	10	(1.1)	..
Adenoma histology‡	..	..	..	..	<0.0001	..	..	..	..	0.0007	..	..	..	..	0.95
Tubular	5430	(75.5)	5708	(79.2)	..	2762	(38.5)	1932	(41.3)	..	684	(37.8)	354	(38.9)	..
Tubulovillous	1149	(16.0)	964	(13.4)	..	3395	(47.4)	2142	(45.7)	..	867	(48.0)	426	(46.8)	..
Villous	106	(1.5)	84	(1.2)	..	683	(9.5)	451	(9.6)	..	217	(12.0)	111	(12.2)	..
Unknown	509	(7.1)	451	(6.3)	..	329	(4.6)	158	(3.4)	..	40	(2.2)	20	(2.2)	..
Adenoma dysplasia‡	..	..	..	..	<0.0001	..	..	..	..	<0.0001	..	..	..	..	0.43
Low-grade	6507	(90.5)	6735	(93.5)	..	5596	(78.1)	3803	(81.2)	..	1340	(74.1)	695	(76.3)	..
High-grade	224	(3.1)	133	(1.8)	..	1236	(17.2)	743	(15.9)	..	420	(23.2)	196	(21.5)	..
Unknown	463	(6.4)	339	(4.7)	..	337	(4.7)	137	(2.9)	..	48	(2.7)	20	(2.2)	..
Proximal polyps	..	..	..	..	0.065	..	..	..	..	0.34	..	..	..	..	0.35
No	4008	(55.7)	4125	(57.2)	..	5016	(70.0)	3238	(69.1)	..	431	(23.8)	232	(25.5)	..
Yes	3186	(44.3)	3082	(42.8)	..	2153	(30.0)	1445	(30.9)	..	1377	(76.2)	679	(74.5)	..
Completeness of colonoscopy	..	..	..	..	<0.0001	..	..	..	..	<0.0001	..	..	..	..	<0.0001
Complete	5570	(77.4)	6149	(85.3)	..	5362	(74.8)	3605	(77.0)	..	1574	(87.1)	780	(85.6)	..
Incomplete	481	(6.7)	659	(9.1)	..	605	(8.4)	716	(15.3)	..	56	(3.1)	67	(7.4)	..
Unknown	1143	(15.9)	399	(5.5)	..	1202	(16.8)	362	(7.7)	..	178	(9.8)	64	(7.0)	..
Bowel preparation quality	..	..	..	..	<0.0001	..	..	..	..	<0.0001	..	..	..	..	<0.0001

Cross AJ, *et al. Gut* 2020; 69:1645–1658. doi: 10.1136/gutjnl-2019-320036

Excellent or good	2562	(35.6)	2583	(35.8)	..	2392	(33.4)	1582	(33.8)	..	768	(42.5)	351	(38.5)	..
Satisfactory	1122	(15.6)	1418	(19.7)	..	995	(13.9)	908	(19.4)	..	246	(13.6)	165	(18.1)	..
Poor	388	(5.4)	580	(8.0)	..	280	(3.9)	380	(8.1)	..	74	(4.1)	69	(7.6)	..
Unknown	3122	(43.4)	2626	(36.4)	..	3502	(48.8)	1813	(38.7)	..	720	(39.8)	326	(35.8)	..
Year of baseline visit	..	..	..	..	<0.0001	..	..	..	..	<0.0001	..	..	..	..	0.021
1984-1999	1126	(15.7)	514	(7.1)	..	1335	(18.6)	535	(11.4)	..	241	(13.3)	88	(9.7)	..
2000-2004	2855	(39.7)	2313	(32.1)	..	2591	(36.1)	1631	(34.8)	..	575	(31.8)	299	(32.8)	..
2005-2010	3213	(44.7)	4380	(60.8)	..	3243	(45.2)	2517	(53.7)	..	992	(54.9)	524	(57.5)	..
Length of baseline visit	..	..	..	..	0.0075	..	..	..	..	0.0001	..	..	..	..	<0.0001
1 day	5688	(79.1)	5666	(78.6)	..	3944	(55.0)	2753	(58.8)	..	773	(42.8)	411	(45.1)	..
2 days-3 months	642	(8.9)	731	(10.1)	..	1428	(19.9)	915	(19.5)	..	334	(18.5)	228	(25.0)	..
3-6 months	469	(6.5)	481	(6.7)	..	897	(12.5)	506	(10.8)	..	316	(17.5)	126	(13.8)	..
≥6 months	395	(5.5)	329	(4.6)	..	900	(12.6)	509	(10.9)	..	385	(21.3)	146	(16.0)	..
Family history of cancer/CRC	..	..	..	..	<0.0001	..	..	..	..	<0.0001	..	..	..	..	<0.0001
No	6106	(84.9)	6830	(94.8)	..	6790	(94.7)	4576	(97.7)	..	1724	(95.4)	897	(98.5)	..
Yes	1088	(15.1)	377	(5.2)	..	379	(5.3)	107	(2.3)	..	84	(4.6)	14	(1.5)	..
Number of hyperplastic polyps	..	..	..	..	<0.0001	..	..	..	..	<0.0001	..	..	..	..	<0.0001
0	5597	(77.8)	5938	(82.4)	..	5783	(80.7)	4010	(85.6)	..	1230	(68.0)	699	(76.7)	..
1	972	(13.5)	818	(11.4)	..	862	(12.0)	433	(9.2)	..	310	(17.1)	129	(14.2)	..
≥2	625	(8.7)	451	(6.3)	..	524	(7.3)	240	(5.1)	..	268	(14.8)	83	(9.1)	..
Any large (≥10mm) hyperplastic	..	..	..	..	..	..	..	..	..	..	..	..	..	..	..
polyp	..	..	..	..	0.39	..	..	..	..	0.0006	..	..	..	..	0.11
No	7120	(99.0)	7143	(99.1)	..	7035	(98.1)	4633	(98.9)	..	1756	(97.1)	894	(98.1)	..
Yes	74	(1.0)	64	(0.9)	..	134	(1.9)	50	(1.1)	..	52	(2.9)	17	(1.9)	..

mm= millimetre. CRC=colorectal cancer.

p-values calculated with χ^2 test to compare patients with and without surveillance.

†Adenoma size was defined according to the largest adenoma seen at baseline.

‡Adenoma histology and dysplasia were defined according to the worst histology (greatest degree of villousness) and worst dysplasia (highest grade) seen at baseline, respectively.

Cross AJ, *et al. Gut* 2020; 69:1645–1658. doi: 10.1136/gutjnl-2019-320036

Supplementary Table 2: Number of surveillance visits, sex, age, year of baseline, and median follow-up time by risk subgroup

	Low-risk group (n=14 401)					Intermediate-risk group (n=11 852)					High-risk group (n=2719)				
	Lower-risk subgroup*		Higher-risk subgroup*			Lower-risk subgroup†		Higher-risk subgroup†			Lower-risk subgroup‡		Higher-risk subgroup‡		
	n	(%)	n	(%)	p-value	n	(%)	n	(%)	p-value	n	(%)	n	(%)	p-value
Total	5235	(36.4)	9166	(63.6)	..	4738	(40.0)	7114	(60.0)	..	1817	(66.8)	902	(33.2)	..
Number of surveillance visits	..	..	..	..	<0.0001	..	..	..	..	<0.0001	..	..	..	..	0.24
0	2804	(53.6)	4403	(48.0)	..	1932	(40.8)	2751	(38.7)	..	606	(33.4)	305	(33.8)	..
1	1432	(27.4)	2527	(27.6)	..	1387	(29.3)	1956	(27.5)	..	474	(26.1)	221	(24.5)	..
2	661	(12.6)	1282	(14.0)	..	930	(19.6)	1349	(19.0)	..	407	(22.4)	186	(20.6)	..
≥3	338	(6.5)	954	(10.4)	..	489	(10.3)	1058	(14.9)	..	330	(18.2)	190	(21.1)	..
Sex	..	..	..	..	0.97	..	..	..	..	0.050	..	..	..	..	0.21
Women	2319	(44.3)	4063	(44.3)	..	2159	(45.6)	3112	(43.7)	..	520	(28.6)	279	(30.9)	..
Men	2916	(55.7)	5103	(55.7)	..	2579	(54.4)	4002	(56.3)	..	1297	(71.4)	623	(69.1)	..
Age, years	..	..	..	..	<0.0001	..	..	..	..	<0.0001	..	..	..	..	0.079
<55	1523	(29.1)	2046	(22.3)	..	984	(20.8)	1113	(15.6)	..	180	(9.9)	103	(11.4)	..
55-64	1452	(27.7)	2539	(27.7)	..	1310	(27.6)	1848	(26.0)	..	514	(28.3)	236	(26.2)	..
65-74	1442	(27.5)	2816	(30.7)	..	1513	(31.9)	2402	(33.8)	..	729	(40.1)	336	(37.3)	..
≥75	818	(15.6)	1765	(19.3)	..	931	(19.6)	1751	(24.6)	..	394	(21.7)	227	(25.2)	..
Year of baseline visit	..	..	..	..	<0.0001	..	..	..	..	<0.0001	..	..	..	..	<0.0001
1984-1999	441	(8.4)	1199	(13.1)	..	630	(13.3)	1240	(17.4)	..	175	(9.6)	154	(17.1)	..
2000-2004	1761	(33.6)	3407	(37.2)	..	1461	(30.8)	2761	(38.8)	..	520	(28.6)	354	(39.2)	..
2005-2010	3033	(57.9)	4560	(49.7)	..	2647	(55.9)	3113	(43.8)	..	1122	(61.8)	394	(43.7)	..
Follow-up time, years (median [IQR])	9.5	(7.3-11.9)	9.6	(7.1-12.6)	0.023¶	9.1	(7.0-11.9)	9.2	(6.1-12.6)	0.98¶	8.3	(6.0-10.6)	8.5	(4.9-12.2)	0.028¶

p-values calculated with χ^2 test to compare patients in the lower-risk and higher-risk subgroups, unless indicated otherwise.

*The higher-risk subgroup included patients with an incomplete colonoscopy or colonoscopy of unknown completeness, a tubulovillous or villous adenoma, or proximal polyps at baseline; the lower-risk subgroup included patients with none of these factors.

†The higher-risk subgroup included patients with an incomplete colonoscopy or colonoscopy of unknown completeness, an adenoma with high-grade dysplasia, or proximal polyps at baseline; the lower-risk subgroup included patients with none of these factors.

‡The higher-risk subgroup included patients with an incomplete colonoscopy or colonoscopy of unknown completeness or an adenoma with high-grade dysplasia at baseline; the lower-risk subgroup included patients with none of these factors.

¶p-values calculated with Wilcoxon rank sum test to compare the lower-risk and higher-risk subgroups.

Cross AJ, *et al. Gut* 2020; 69:1645–1658. doi: 10.1136/gutjnl-2019-320036

Supplementary Table 3: Long-term colorectal cancer incidence in the low-risk group by baseline characteristics and number of surveillance visits, including only patients with a complete baseline colonoscopy

	n	(%)	Person -years	CRC cases	Incidence per 100,000 person years (95% CI)		Univariable HR (95% CI)	p-value	Multivariable HR (95% CI)	p-value
Total	11,719	(100)	108,319	144	133	(113-157)	..	..	..	..
Number of surveillance visits*	..	..	..	..	..	..	..	<0.0001	..	0.0001
0	6149	(52.5)	69,666	108	155	(128-187)	1	..	1	..
1	3222	(27.5)	25,706	29	113	(78-162)	0.54 (0.35-0.82)	..	0.56 (0.36-0.85)	..
2	1515	(12.9)	9055	5	55	(23-133)	0.22 (0.09-0.56)	..	0.24 (0.10-0.61)	..
≥3	833	(7.1)	3893	2	51	(13-205)	0.18 (0.04-0.77)	..	0.20 (0.05-0.83)	..
Sex	..	..	..	..	..	..	..	1.00	..	0.70
Women	5128	(43.8)	48,698	65	133	(105-170)	1	..	1	..
Men	6591	(56.2)	59,621	79	133	(106-165)	1.00 (0.72-1.39)	..	1.07 (0.77-1.48)	..
Age, years	..	..	..	..	..	..	..	<0.0001	..	<0.0001
<55	2852	(24.3)	30,666	14	46	(27-77)	1	..	1	..
55-64	3268	(27.9)	32,845	35	107	(77-148)	2.37 (1.28-4.41)	..	2.27 (1.22-4.22)	..
65-74	3487	(29.8)	30,653	60	196	(152-252)	4.50 (2.51-8.07)	..	4.00 (2.23-7.17)	..
≥75	2112	(18.0)	14,156	35	247	(178-344)	6.10 (3.27-11.39)	..	4.92 (2.62-9.22)	..
Number of adenomas	..	..	..	..	..	..	..	0.21	..	0.55
1	9554	(81.5)	88,674	112	126	(105-152)	1	..	1	..
2	2165	(18.5)	19,646	32	163	(115-230)	1.30 (0.88-1.92)	..	1.13 (0.76-1.69)	..
Adenoma histology†	..	..	..	..	..	..	..	0.066	..	0.060
Tubular	9253	(79.0)	85,190	103	121	(100-147)	1	..	1	..
Tubulovillous	1587	(13.5)	14,364	30	209	(146-299)	}1.65 (1.10-2.46)	..	}1.67 (1.11-2.49)	..
Villous	124	(1.1)	1230	1	81	(11-577)		..		..
Unknown	755	(6.4)	7536	10	133	(71-247)	1.07 (0.56-2.06)	..	1.15 (0.60-2.21)	..
Adenoma dysplasia†	..	..	..	..	..	..	..	0.36	..	0.38
Low-grade	10,993	(93.8)	100,640	132	131	(111-156)	1	..	1	..
High-grade	262	(2.2)	2424	6	247	(111-551)	1.86 (0.82-4.21)	..	1.85 (0.81-4.25)	..
Unknown	464	(4.0)	5255	6	114	(51-254)	0.84 (0.37-1.90)	..	0.87 (0.38-2.00)	..
Proximal polyps	..	..	..	..	..	..	..	0.0025	..	0.0050
No	6251	(53.3)	59,177	61	103	(80-132)	1	..	1	..
Yes	5468	(46.7)	49,142	83	169	(136-209)	1.66 (1.19-2.31)	..	1.61 (1.15-2.24)	..
Bowel preparation quality	..	..	..	..	..	..	..	0.058	..	0.11
Excellent or good	3998	(34.1)	38,324	66	172	(135-219)	1	..	1	..
Satisfactory	2294	(19.6)	19,860	26	131	(89-192)	0.77 (0.49-1.22)	..	0.74 (0.47-1.17)	..
Poor	737	(6.3)	5958	7	117	(56-246)	0.70 (0.32-1.52)	..	0.69 (0.31-1.49)	..
Unknown	4690	(40.0)	44,177	45	102	(76-136)	0.59 (0.41-0.87)	..	0.63 (0.43-0.92)	..
Year of baseline visit	..	..	..	..	..	..	..	0.93	..	0.80
1984-1994	941	(8.0)	12,859	17	132	(82-213)	1	..	1	..
2000-2004	3839	(32.8)	41,000	57	139	(107-180)	1.09 (0.61-1.95)	..	0.88 (0.49-1.57)	..
2005-2010	6939	(59.2)	54,460	70	129	(102-162)	1.12 (0.62-2.03)	..	0.81 (0.45-1.48)	..
Length of baseline visit	..	..	..	..	..	..	..	0.54	..	0.51
1 day	9214	(78.6)	85,093	112	132	(109-158)	1	..	1	..
2 days-3 months	1118	(9.5)	9808	16	163	(100-266)	1.26 (0.74-2.12)	..	1.33 (0.78-2.27)	..
3-6 months	781	(6.7)	7455	11	148	(82-266)	1.11 (0.60-2.07)	..	1.27 (0.68-2.38)	..
≥6 months	606	(5.2)	5963	5	84	(35-201)	0.63 (0.26-1.54)	..	0.71 (0.29-1.74)	..

CI=confidence interval. CRC=colorectal cancer. HR=hazard ratio.

The multivariable model contained number of surveillance visits, age, adenoma histology, and proximal polyps, the variables selected in the final multivariable model for all low-risk patients in the main analysis (except for colonoscopy completeness), as presented in Table 1. For these variables, the multivariable HRs were from the multivariable model and the p-values were for inclusion of the variable in the model, calculated with the likelihood ratio test. For the remaining variables, the multivariable HRs were for if the variable was added as an additional variable to the multivariable model.

*The number of surveillance visits was included as a time-varying covariate; patients who attended any surveillance contributed person-years to more than one category of number of surveillance visits.

Cross AJ, *et al. Gut* 2020; 69:1645–1658. doi: 10.1136/gutjnl-2019-320036

†Adenoma histology and dysplasia were defined according to the worst histology (greatest degree of villousness) and worst dysplasia (highest grade) seen at baseline, respectively.

Cross AJ, *et al. Gut* 2020; 69:1645–1658. doi: 10.1136/gutjnl-2019-320036

Supplementary Table 4: Long-term colorectal cancer incidence in the intermediate-risk group by baseline characteristics and number of surveillance visits, including only patients with a complete baseline colonoscopy

	n	(%)	Person -years	CRC case s	Incidence per 100,000 person years (95% CI)		Univariable HR (95% CI)	p-value	Multivariable HR (95% CI)	p-value
Total	8967	(100)	80,572	150	186	(159-218)	..	..	..	..
Number of surveillance visits*	..	..	..	..	..	..	..	0.0041	..	0.0080
0	3605	(40.2)	40,921	85	208	(168-257)	1	..	1	..
1	2565	(28.6)	24,087	41	170	(125-231)	0.63 (0.43-0.93)	..	0.64 (0.43-0.95)	..
2	1755	(19.6)	10,462	16	153	(94-250)	0.47 (0.27-0.83)	..	0.50 (0.28-0.88)	..
≥3	1042	(11.6)	5103	8	157	(78-313)	0.36 (0.17-0.78)	..	0.36 (0.16-0.79)	..
Sex	..	..	..	..	..	..	..	0.80	..	0.38
Women	3874	(43.2)	36,048	66	183	(144-233)	1	..	1	..
Men	5093	(56.8)	44,524	84	189	(152-234)	1.04 (0.76-1.44)	..	1.16 (0.83-1.61)	..
Age, years	..	..	..	..	..	..	..	<0.0001	..	<0.0001
<55	1573	(17.5)	17,584	18	102	(64-162)	1	..	1	..
55-64	2416	(26.9)	24,093	25	104	(70-154)	1.06 (0.58-1.94)	..	1.04 (0.57-1.91)	..
65-74	3013	(33.6)	26,134	63	241	(188-309)	2.64 (1.56-4.47)	..	2.52 (1.48-4.28)	..
≥75	1965	(21.9)	12,761	44	345	(257-463)	4.13 (2.36-7.21)	..	3.49 (1.99-6.14)	..
Number of adenomas	..	..	..	..	..	..	..	0.24	..	0.40
1	5665	(63.2)	52,222	92	176	(144-216)	1	..	1	..
2	2461	(27.5)	21,306	48	225	(170-299)	1.31 (0.92-1.86)	..	1.05 (0.73-1.52)	..
3 or 4	841	(9.4)	7044	10	142	(76-264)	0.84 (0.44-1.62)	..	0.67 (0.33-1.32)	..
Adenoma size, mm†	..	..	..	..	..	..	..	0.21	..	0.21
<10	841	(9.4)	7044	10	142	(76-264)	1	..	1	..
10-19	5160	(57.5)	46,777	80	171	(137-213)	1.16 (0.60-2.24)	..	1.43 (0.73-2.83)	..
≥20	2966	(33.1)	26,751	60	224	(174-289)	1.52 (0.78-2.98)	..	1.77 (0.87-3.57)	..
Adenoma histology‡	..	..	..	..	..	..	..	<0.0001	..	0.0003
Tubular	3660	(40.8)	33,091	41	124	(91-168)	1	..	1	..
Tubulovillous	4204	(46.9)	37,334	76	204	(163-255)	1.67 (1.14-2.44)	..	1.62 (1.10-2.38)	..
Villous	768	(8.6)	6812	15	220	(133-365)	1.80 (1.00-3.25)	..	1.39 (0.75-2.56)	..
Unknown	335	(3.7)	3334	18	540	(340-857)	4.17 (2.39-7.28)	..	4.28 (2.27-8.08)	..
Adenoma dysplasia‡	..	..	..	..	..	..	..	0.11	..	0.20
Low-grade	7251	(80.9)	64,995	111	171	(142-206)	1	..	1	..
High-grade	1473	(16.4)	12,621	29	230	(160-331)	1.36 (0.90-2.05)	..	1.23 (0.81-1.87)	..
Unknown	243	(2.7)	2956	10	338	(182-629)	1.82 (0.95-3.49)	..	1.80 (0.92-3.50)	..
Proximal polyps	..	..	..	..	..	..	..	0.0009	..	0.0007
No	5781	(64.5)	53,627	81	151	(121-188)	1	..	1	..
Yes	3186	(35.5)	26,945	69	256	(202-324)	1.74 (1.26-2.40)	..	1.77 (1.28-2.45)	..
Bowel preparation quality	..	..	..	..	..	..	..	0.11	..	0.093
Excellent or good	3046	(34.0)	27,581	46	167	(125-223)	1	..	1	..
Satisfactory	1664	(18.6)	13,640	28	205	(142-297)	1.27 (0.79-2.03)	..	1.34 (0.83-2.14)	..
Poor	454	(5.1)	3320	12	361	(205-636)	2.24 (1.19-4.23)	..	2.00 (1.05-3.78)	..
Unknown	3803	(42.4)	36,031	64	178	(139-227)	1.02 (0.70-1.50)	..	0.92 (0.62-1.37)	..
Year of baseline visit	..	..	..	..	..	..	..	0.061	..	0.020
1984-1999	1044	(11.6)	13,531	41	303	(223-412)	1	..	1	..
2000-2004	2805	(31.3)	28,734	53	184	(141-241)	0.63 (0.41-0.96)	..	0.55 (0.36-0.86)	..
2005-2010	5118	(57.1)	38,307	56	146	(113-190)	0.59 (0.37-0.93)	..	0.54 (0.33-0.86)	..
Length of baseline visit	..	..	..	..	..	..	..	0.048	..	0.019
1 day	5040	(56.2)	44,805	67	150	(118-190)	1	..	1	..
2 days-3 months	1766	(19.7)	16,001	36	225	(162-312)	1.52 (1.01-2.27)	..	1.75 (1.16-2.64)	..
3-6 months	1083	(12.1)	10,070	21	209	(136-320)	1.39 (0.85-2.27)	..	1.44 (0.88-2.37)	..
≥6 months	1078	(12.0)	9695	26	268	(183-394)	1.77 (1.13-2.79)	..	1.79 (1.12-2.85)	..

CI=confidence interval. CRC=colorectal cancer. HR=hazard ratio. mm= millimetre.

The multivariable model contained number of surveillance visits, age, adenoma dysplasia, proximal polyps, year of baseline visit, and length of baseline visit, the variables selected in the final multivariable model for all intermediate-risk patients in the main analysis (except for colonoscopy completeness), as presented in Table 2.

Cross AJ, *et al. Gut* 2020; 69:1645–1658. doi: 10.1136/gutjnl-2019-320036

For these variables, the multivariable HRs were from the multivariable model and the p-values were for inclusion of the variable in the model, calculated with the likelihood ratio test. For the remaining variables, the multivariable HRs were for if the variable was added as an additional variable to the multivariable model.
*The number of surveillance visits was included as a time-varying covariate; patients who attended any surveillance contributed person-years to more than one category of number of surveillance visits.
†Adenoma size was defined according to the largest adenoma seen at baseline.
‡Adenoma histology and dysplasia were defined according to the worst histology (greatest degree of villousness) and worst dysplasia (highest grade) seen at baseline, respectively.

Cross AJ, *et al. Gut* 2020; 69:1645–1658. doi: 10.1136/gutjnl-2019-320036

Supplementary Table 5: Long-term colorectal cancer incidence in the high-risk group by baseline characteristics and number of surveillance visits, including only patients with a complete baseline colonoscopy

	n	(%)	Person-years	CRC cases	Incidence per 100,000 person years (95% CI)		Univariable HR (95% CI)	p-value	Multivariable HR (95% CI)	p-value
Total	2354	(100)	19,266	64	332	(260-424)	..	..	..	..
Number of surveillance visits*	..	..	..	..	..	..	..	0.012	..	0.0069
0	780	(33.1)	7847	33	421	(299-592)	1	..	1	..
1	623	(26.5)	6181	19	307	(196-482)	0.51 (0.29-0.91)	..	0.50 (0.28-0.89)	..
2	518	(22.0)	3303	7	212	(101-445)	0.30 (0.13-0.70)	..	0.28 (0.12-0.66)	..
≥3	433	(18.4)	1935	5	258	(108-621)	0.40 (0.14-1.09)	..	0.37 (0.14-1.03)	..
Sex	..	..	..	..	..	..	..	0.72	..	0.81
Women	669	(28.4)	5648	20	354	(228-549)	1	..	1	..
Men	1685	(71.6)	13,618	44	323	(240-434)	0.91 (0.54-1.54)	..	0.94 (0.55-1.59)	..
Age, years	..	..	..	..	..	..	..	0.045	..	0.20
<55	224	(9.5)	2445	4	164	(61-436)	1	..	1	..
55-64	655	(27.8)	5915	15	254	(153-421)	1.56 (0.52-4.70)	..	1.59 (0.53-4.79)	..
65-74	942	(40.0)	7580	27	356	(244-519)	2.21 (0.77-6.34)	..	2.13 (0.74-6.11)	..
≥75	533	(22.6)	3325	18	541	(341-859)	3.45 (1.16-10.25)	..	2.70 (0.90-8.05)	..
Number of adenomas	..	..	..	..	..	..	..	0.89	..	0.81
3	1038	(44.1)	8736	27	309	(212-451)	1	..	1	..
4	496	(21.1)	4016	13	324	(188-557)	1.05 (0.54-2.04)	..	1.09 (0.56-2.12)	..
5	400	(17.0)	3180	13	409	(237-704)	1.31 (0.67-2.53)	..	1.39 (0.72-2.71)	..
≥6	420	(17.8)	3333	11	330	(183-596)	1.07 (0.53-2.16)	..	1.17 (0.57-2.37)	..
Adenoma size, mm†	..	..	..	..	..	..	..	0.38	..	0.78
<10	225	(9.6)	1893	5	264	(110-635)	1	..	1	..
10-19	1168	(49.6)	9566	28	293	(202-424)	1.12 (0.43-2.90)	..	0.99 (0.38-2.57)	..
≥20	947	(40.2)	7683	31	403	(284-574)	1.55 (0.60-3.98)	..	1.19 (0.45-3.14)	..
Unknown	14	(0.6)	123	0	0	NA	NA	..	NA	..
Adenoma histology‡	..	..	..	..	..	..	..	0.24	..	0.49
Tubular	886	(37.6)	7379	22	298	(196-453)	1	..	1	..
Tubulovillous	1151	(48.9)	9278	30	323	(226-462)	1.10 (0.63-1.91)	..	0.99 (0.57-1.73)	..
Villous	277	(11.8)	2197	12	546	(310-962)	1.85 (0.92-3.74)	..	1.49 (0.72-3.07)	..
Unknown	40	(1.7)	411	0	0	NA	NA	..	NA	..
Adenoma dysplasia‡	..	..	..	..	..	..	..	0.0010	..	0.0005
Low-grade	1783	(75.7)	14,680	38	259	(188-356)	1	..	1	..
High-grade	537	(22.8)	4234	26	614	(418-902)	2.40 (1.46-3.95)	..	2.51 (1.52-4.14)	..
Unknown	34	(1.4)	352	0	0	NA	NA	..	NA	..
Proximal polyps	..	..	..	..	..	..	..	0.29	..	0.18
No	465	(19.8)	3982	10	251	(135-467)	1	..	1	..
Yes	1889	(80.3)	15,284	54	353	(271-461)	1.42 (0.72-2.79)	..	1.56 (0.79-3.07)	..
Bowel preparation quality	..	..	..	..	..	..	..	0.64	..	0.68
Excellent or good	935	(39.7)	7922	24	303	(203-452)	1	..	1	..
Satisfactory	385	(16.4)	2903	10	344	(185-640)	1.13 (0.54-2.37)	..	0.99 (0.47-2.07)	..
Poor	129	(5.5)	852	5	587	(244-1410)	1.99 (0.76-5.21)	..	1.88 (0.71-4.95)	..
Unknown	905	(38.5)	7588	25	329	(223-488)	1.11 (0.64-1.95)	..	1.09 (0.62-1.91)	..
Year of baseline visit	..	..	..	..	..	..	..	0.56	..	0.69
1984-1999	221	(9.4)	2610	5	192	(80-460)	1	..	1	..
2000-2004	685	(29.1)	6383	24	376	(252-561)	1.64 (0.63-4.31)	..	1.50 (0.57-3.94)	..
2005-2010	1448	(61.5)	10,273	35	341	(245-475)	1.54 (0.59-4.01)	..	1.41 (0.54-3.70)	..
Length of baseline visit	..	..	..	..	..	..	..	0.79	..	0.96
1 day	1021	(43.4)	8406	25	297	(201-440)	1	..	1	..
2 days-3 months	471	(20.0)	3662	13	355	(206-611)	1.20 (0.61-2.35)	..	1.04 (0.53-2.04)	..
3-6 months	389	(16.5)	3214	10	311	(167-578)	1.05 (0.50-2.18)	..	0.95 (0.45-2.00)	..
≥6 months	473	(20.1)	3984	16	402	(246-656)	1.37 (0.73-2.57)	..	1.15 (0.60-2.20)	..

CI=confidence interval. CRC=colorectal cancer. HR=hazard ratio. mm= millimetre. NA=not applicable.
All patients with unknown adenoma dysplasia were excluded from the multivariable model as no events occurred in these patients. The multivariable model contained number of surveillance visits and adenoma

dysplasia, the variables selected in the final multivariable model for all high-risk patients in the main analysis (except for colonoscopy completeness), as presented in Table 3. For these variables, the multivariable HRs were from the multivariable model and the p-values were for inclusion of the variable in the model, calculated with the likelihood ratio test. For the remaining variables, the multivariable HRs were for if the variable was added as an additional variable to the multivariable model.
*The number of surveillance visits was included as a time-varying covariate; patients who attended any surveillance contributed person-years to more than one category of number of surveillance visits.
†Adenoma size was defined according to the largest adenoma seen at baseline.
‡Adenoma histology and dysplasia were defined according to the worst histology (greatest degree of villousness) and worst dysplasia (highest grade) seen at baseline, respectively.

Cross AJ, *et al. Gut* 2020; 69:1645–1658. doi: 10.1136/gutjnl-2019-320036

Supplementary Table 6: Incidence of colorectal cancer and unadjusted effect of surveillance on colorectal cancer incidence by number of surveillance visits, including only patients with a complete baseline colonoscopy

								Effect of surveillance*	
		n	(%)	Person-years	CRC cases	Incidence per 100,000 person-years (95% CI)		Univariable HR (95% CI)	p-value
**Low-risk group**	Whole risk group	..	..	..	..	..	..	..	<0.0001
	0 visits	6149	(52.5)	69,666	108	155	(128-187)	1	..
	1 visit	3222	(27.5)	25,706	29	113	(78-162)	0.54 (0.35-0.82)	..
	≥2 visits	2348	(20.0)	12,948	7	54	(26-113)	0.21 (0.10-0.47)	..
	Total	11,719	(100)	108,319	144	133	(113-157)	..	..
	Lower-risk subgroup†	..	..	..	..	..	..	..	0.15
	0 visits	2804	(53.6)	32,903	31	94	(66-134)	1	..
	1 visit	1432	(27.4)	11,410	8	70	(35-140)	0.54 (0.25-1.20)	..
	≥2 visits	999	(19.1)	5472	3	55	(18-170)	0.42 (0.12-1.48)	..
	Total	5235	(44.7)	49,785	42	84	(62-114)	..	..
	Higher-risk subgroup†	..	..	..	..	..	..	..	<0.0001
	0 visits	3345	(51.6)	36,763	77	209	(168-262)	1	..
	1 visit	1790	(27.6)	14,296	21	147	(96-225)	0.50 (0.31-0.83)	..
	≥2 visits	1349	(20.8)	7475	4	54	(20-143)	0.14 (0.05-0.39)	..
	Total	6484	(55.3)	58,534	102	174	(144-212)	..	..
**Intermediate-risk group**	Whole risk group	..	..	..	..	..	..	..	0.0016
	0 visits	3605	(40.2)	40,921	85	208	(168-257)	1	..
	1 visit	2565	(28.6)	24,087	41	170	(125-231)	0.63 (0.43-0.93)	..
	≥2 visits	2797	(31.2)	15,565	24	154	(103-230)	0.43 (0.26-0.71)	..
	Total	8967	(100)	80,572	150	186	(159-218)	..	..
	Lower-risk subgroup‡	..	..	..	..	..	..	..	0.30
	0 visits	1932	(40.8)	23,237	33	142	(101-200)	1	..
	1 visit	1387	(29.3)	13,151	16	122	(75-199)	0.66 (0.35-1.23)	..
	≥2 visits	1419	(30.0)	8082	13	161	(93-277)	0.63 (0.31-1.29)	..
	Total	4738	(52.8)	44,470	62	139	(109-179)	..	..
	Higher-risk subgroup‡	..	..	..	..	..	..	..	0.0006
	0 visits	1673	(39.6)	17,684	52	294	(224-386)	1	..
	1 visit	1178	(27.9)	10,936	25	229	(154-338)	0.58 (0.35-0.96)	..
	≥2 visits	1378	(32.6)	7482	11	147	(81-265)	0.28 (0.14-0.56)	..
	Total	4229	(47.2)	36,102	88	244	(198-300)	..	..
**High-risk group**	Whole risk group	..	..	..	..	..	..	..	0.0046
	0 visits	780	(33.1)	7847	33	421	(299-592)	1	..
	1 visit	623	(26.5)	6181	19	307	(196-482)	0.51 (0.29-0.91)	..
	≥2 visits	951	(40.4)	5238	12	229	(130-403)	0.33 (0.16-0.68)	..
	Total	2354	(100)	19,266	64	332	(260-424)	..	..
	Lower-risk subgroup§	..	..	..	..	..	..	..	0.26
	0 visits	606	(33.4)	6226	17	273	(170-439)	1	..
	1 visit	474	(26.1)	4766	12	252	(143-443)	0.66 (0.31-1.41)	..
	≥2 visits	737	(40.6)	4039	9	223	(116-428)	0.49 (0.20-1.18)	..
	Total	1817	(77.2)	15,032	38	253	(184-347)	..	..
	Higher-risk subgroup§	..	..	..	..	..	..	..	0.0030
	0 visits	174	(32.4)	1620	16	987	(605-1612)	1	..
	1 visit	149	(27.8)	1414	7	495	(236-1038)	0.34 (0.14-0.85)	..
	≥2 visits	214	(39.9)	1199	3	250	(81-776)	0.15 (0.04-0.56)	..
	Total	537	(22.8)	4234	26	614	(418-902)	..	..

CI=confidence interval. CRC=colorectal cancer. HR=hazard ratio.

p-values calculated with the likelihood ratio test.

*The number of surveillance visits was included as a time-varying covariate; patients who attended any surveillance contributed person-years to more than one category of number of surveillance visits.

†The higher-risk subgroup included patients with a tubulovillous or villous adenoma, or proximal polyps at baseline; the lower-risk subgroup included patients with none of these factors.

‡The higher-risk subgroup included patients with an adenoma with high-grade dysplasia, or proximal polyps at baseline; the lower-risk subgroup included patients with none of these factors.

§The higher-risk subgroup included patients with an adenoma with high-grade dysplasia at baseline; the lower-risk subgroup included patients with none of these factors.

Cross AJ, *et al. Gut* 2020; 69:1645–1658. doi: 10.1136/gutjnl-2019-320036

Supplementary Table 7: Cumulative colorectal cancer incidence and age- and sex-standardised incidence ratios, including only patients with a complete baseline colonoscopy

							Follow-up									
							3 years		5 years		10 years			Standardisation		
	n	(%)	Person -years	CRC cases	Incidence per 100,000 person-years (95% CI)		CRC cases	Cumulative incidence (%)*	CRC cases	Cumulative incidence (%)*	CRC cases	Cumulative incidence (%)*	p-value	Expected CRCs†	SIR (95% CI)	
**Low-risk group**	..	..	..	..	..	..	..	..	..	..	..	..	..	..	..	..
After baseline (no surveillance, censored at first surveillance)	..	..	..	..	..	..	..	..	..	..	..	..	<0.0001	..	..	..
Whole risk group	11,719	(100)	69,666	108	155	(128-187)	29	0.3% (0.2-0.4)	52	0.6% (0.5-0.8)	92	1.6% (1.3-2.1)	..	137	0.79	(0.65-0.95)
Lower-risk subgroup‡	5235	(45)	32,903	31	94	(66-134)	6	0.1% (0.1-0.3)	11	0.3% (0.2-0.5)	29	1.2% (0.8-1.7)	..	60	0.51	(0.35-0.73)
Higher-risk subgroup‡	6484	(55)	36,763	77	209	(168-262)	23	0.4% (0.3-0.7)	41	0.9% (0.7-1.2)	63	2.1% (1.6-2.7)	..	77	1.00	(0.79-1.26)
After first surveillance (one surveillance visit, censored at second surveillance)	..	..	..	..	..	..	..	..	..	..	..	..	0.066	..	..	..
Whole risk group	5570	(100)	25,706	29	113	(78-162)	12	0.3% (0.2-0.5)	20	0.5% (0.3-0.8)	28	1.4% (0.8-2.4)	..	51	0.57	(0.38-0.82)
Lower-risk subgroup‡	2431	(44)	11,410	8	70	(35-140)	5	0.2% (0.1-0.6)	7	0.4% (0.2-0.8)	8	0.6% (0.2-1.4)	..	21	0.38	(0.16-0.74)
Higher-risk subgroup‡	3139	(56)	14,296	21	147	(96-225)	7	0.3% (0.1-0.6)	13	0.6% (0.4-1.1)	20	2.1% (1.1-3.9)	..	30	0.70	(0.44-1.08)
After second surveillance (two or more surveillance visits, censored at end of follow-up)	..	..	..	..	..	..	..	..	..	..	..	..	0.97	..	..	..
Whole risk group	2348	(100)	12,948	7	54	(26-113)	0	-	1	0.1% (0.0-0.6)	5	0.8% (0.3-1.9)	..	27	0.26	(0.10-0.53)
Lower-risk subgroup‡	999	(43)	5472	3	55	(18-170)	0	-	0	-	2	0.7% (0.2-3.0)	..	10	0.29	(0.06-0.84)
Higher-risk subgroup‡	1349	(57)	7475	4	54	(20-143)	0	-	1	0.2% (0.0-1.1)	3	0.8% (0.2-2.6)	..	17	0.24	(0.07-0.62)
**Intermediate-risk group**	..	..	..	..	..	..	..	..	..	..	..	..	..	..	..	..
After baseline (no surveillance, censored at first surveillance)	..	..	..	..	..	..	..	..	..	..	..	..	0.0004	..	..	..
Whole risk group	8967	(100)	40,921	85	208	(168-257)	35	0.5% (0.4-0.7)	55	1.1% (0.8-1.4)	74	2.1% (1.6-2.7)	..	88	0.97	(0.77-1.19)
Lower-risk subgroup§	4738	(53)	23,237	33	142	(101-200)	13	0.4% (0.2-0.6)	20	0.7% (0.4-1.1)	27	1.3% (0.8-2.1)	..	47	0.70	(0.48-0.99)
Higher-risk subgroup§	4229	(47)	17,684	52	294	(224-386)	22	0.7% (0.5-1.1)	35	1.5% (1.1-2.2)	47	3.1% (2.2-4.4)	..	41	1.27	(0.95-1.66)
After first surveillance (one surveillance visit, censored at second surveillance)	..	..	..	..	..	..	..	..	..	..	..	..	0.028	..	..	..
Whole risk group	5362	(100)	24,087	41	170	(125-231)	10	0.2% (0.1-0.4)	22	0.8% (0.5-1.2)	39	2.9% (2.0-4.3)	..	53	0.77	(0.55-1.05)
Lower-risk subgroup§	2806	(52)	13,151	16	122	(75-199)	3	0.1% (0.0-0.4)	8	0.5% (0.2-1.0)	15	1.9% (1.0-3.4)	..	27	0.59	(0.34-0.96)

Cross AJ, *et al. Gut* 2020; 69:1645–1658. doi: 10.1136/gutjnl-2019-320036

Higher-risk subgroup§	2556	(48)	10,936	25	229	(154-338)	7	0.4% (0.2-0.7)	14	1.1% (0.6-1.8)	24	4.3% (2.6-7.3)	..	26	0.96	(0.62-1.42)
After second surveillance (two or more surveillance visits, censored at end of follow-up)	..	..	..	..	..	..	..	..	..	..	..	..	0.99	..	..	..
Whole risk group	2797	(100)	15,565	24	154	(103-230)	6	0.3% (0.1-0.6)	10	0.5% (0.3-1.0)	18	1.7% (1.0-2.8)	..	36	0.66	(0.42-0.98)
Lower-risk subgroup§	1419	(51)	8082	13	161	(93-277)	3	0.3% (0.1-0.8)	6	0.6% (0.3-1.5)	10	1.7% (0.8-3.3)	..	18	0.72	(0.38-1.22)
Higher-risk subgroup§	1378	(49)	7482	11	147	(81-265)	3	0.3% (0.1-0.8)	4	0.4% (0.1-1.0)	8	1.7% (0.8-3.7)	..	18	0.61	(0.30-1.09)
**High-risk group**	..	..	..	..	..	..	..	..	..	..	..	..	..	..	..	..
After baseline (no surveillance, censored at first surveillance)	..	..	..	..	..	..	..	..	..	..	..	..	<0.0001	..	..	..
Whole risk group	2354	(100)	7847	33	421	(299-592)	13	0.9% (0.5-1.6)	23	2.6% (1.7-4.0)	32	5.8% (3.8-8.9)	..	20	1.68	(1.15-2.35)
Lower-risk subgroup¶	1817	(77)	6226	17	273	(170-439)	7	0.6% (0.3-1.4)	13	1.9% (1.0-3.4)	17	3.8% (2.1-6.8)	..	15	1.10	(0.64-1.76)
Higher-risk subgroup¶	537	(23)	1620	16	987	(605-1612)	6	2.1% (0.9-4.8)	10	5.4% (2.7-10.4)	15	14.4% (7.8-25.7)	..	4	3.76	(2.15-6.11)
After first surveillance (one surveillance visit, censored at second surveillance)	..	..	..	..	..	..	..	..	..	..	..	..	0.12	..	..	..
Whole risk group	1574	(100)	6181	19	307	(196-482)	8	0.6% (0.3-1.3)	15	2.0% (1.2-3.5)	18	4.4% (2.1-8.9)	..	16	1.21	(0.73-1.89)
Lower-risk subgroup¶	1211	(77)	4766	12	252	(143-443)	5	0.5% (0.2-1.3)	8	1.3% (0.6-2.9)	11	4.4% (1.8-10.6)	..	12	1.01	(0.52-1.76)
Higher-risk subgroup¶	363	(23)	1414	7	495	(236-1038)	3	1.1% (0.3-3.2)	7	4.5% (2.0-9.7)	7	4.5% (2.0-9.7)	..	4	1.82	(0.73-3.75)
After second surveillance (two or more surveillance visits, censored at end of follow-up)	..	..	..	..	..	..	..	..	..	..	..	..	0.91	..	..	..
Whole risk group	951	(100)	5238	12	229	(130-403)	2	0.3% (0.1-1.0)	7	1.1% (0.5-2.3)	12	2.8% (1.5-5.2)	..	14	0.85	(0.44-1.48)
Lower-risk subgroup¶	737	(77)	4039	9	223	(116-428)	1	0.2% (0.0-1.2)	6	1.3% (0.6-2.8)	9	2.4% (1.2-4.7)	..	11	0.83	(0.38-1.58)
Higher-risk subgroup¶	214	(23)	1199	3	250	(81-776)	1	0.5% (0.1-3.6)	1	0.5% (0.1-3.6)	3	3.7% (1.0-13.2)	..	3	0.88	(0.18-2.58)

CI=confidence interval. CRC=colorectal cancer. SIR=standardised incidence ratio.

p-values calculated with the log-rank test to compare incidence in the lower-risk and higher-risk subgroups of each risk group.

*One minus the Kaplan-Meier estimator of the survival function was used to estimate the cumulative incidence of colorectal cancer.

†Expected numbers of colorectal cancers were calculated by multiplying the sex and five-year age-group-specific observed person-years by the corresponding incidence rates in the general population of England in 2007.

‡The higher-risk subgroup included patients with a tubulovillous or villous adenoma, or proximal polyps at baseline; the lower-risk subgroup included patients with none of these factors.

§The higher-risk subgroup included patients with an adenoma with high-grade dysplasia, or proximal polyps at baseline; the lower-risk subgroup included patients with none of these factors.

¶The higher-risk subgroup included patients with an adenoma with high-grade dysplasia at baseline; the lower-risk subgroup included patients with none of these factors.
